# Proteomic profiling of extracellular vesicles allows for human breast cancer subtyping

**DOI:** 10.1038/s42003-019-0570-8

**Published:** 2019-09-03

**Authors:** Stamatia Rontogianni, Eleni Synadaki, Bohui Li, Marte C. Liefaard, Esther H. Lips, Jelle Wesseling, Wei Wu, Maarten Altelaar

**Affiliations:** 1 Biomolecular Mass Spectrometry and Proteomics Group, Utrecht Institute for Pharmaceutical Science, Utrecht University, Utrecht, The Netherlands; 2 Netherlands Proteomics Center, Padualaan 8, 3584 CH Utrecht, The Netherlands; 3 Division of Molecular Pathology, The Netherlands Cancer Institute, 1066 CX Amsterdam, The Netherlands; 4 Department of Pathology, The Netherlands Cancer Institute, 1066 CX Amsterdam, The Netherlands; 5 Mass Spectrometry and Proteomics Facility, The Netherlands Cancer Institute, 1066 CX Amsterdam, The Netherlands

**Keywords:** Breast cancer, Mass spectrometry, Proteomic analysis

## Abstract

Extracellular vesicles (EVs) are a potential source of disease-associated biomarkers for diagnosis. In breast cancer, comprehensive analyses of EVs could yield robust and reliable subtype-specific biomarkers that are still critically needed to improve diagnostic routines and clinical outcome. Here, we show that proteome profiles of EVs secreted by different breast cancer cell lines are highly indicative of their respective molecular subtypes, even more so than the proteome changes within the cancer cells. Moreover, we detected molecular evidence for subtype-specific biological processes and molecular pathways, hyperphosphorylated receptors and kinases in connection with the disease, and compiled a set of protein signatures that closely reflect the associated clinical pathophysiology. These unique features revealed in our work, replicated in clinical material, collectively demonstrate the potential of secreted EVs to differentiate between breast cancer subtypes and show the prospect of their use as non-invasive liquid biopsies for diagnosis and management of breast cancer patients.

## Abstract (web summary)

Stamatia Rontogianni et al. show that the proteomic profiles of extracellular vesicles secreted by different classes of breast cancer cell lines can be used as biomarkers of their respective subtype. They show these provide molecular evidence for subtype-specific biological processes and pathways.

## Introduction

In the last decade, circulating exosomes and/or microvesicles, collectively termed extracellular vesicles (EVs), have attracted great interest as a mode of intercellular communication in both physiological and pathological conditions^1^. EVs are heterogeneous populations of nano-sized cell-derived membrane vesicles that are constantly released by a variety of cell types into the extracellular environment. These vesicles transfer proteins, lipids, and nucleic acids from a cell of origin to recipient cells and play a crucial role in cell-to-cell communication^2^. Importantly, EVs are present in accessible biofluids such as blood, urine, saliva, and breast milk, and reflect a molecular fingerprint of the releasing cell type, enabling a molecular readout of practically all organs in the body^3,4^. For instance, cancer-derived EVs have been shown to contain tumor-specific molecules that promote cancer progression, invasion and metastasis, remodeling of the tumor microenvironment and angiogenesis^1,5–7^. In further support of paracrine signaling function, a large body of evidence suggests that cancer cells secrete significantly more EVs than non-cancerous cells^8^. Thus, profiling EV contents directly from patient-derived body fluids could provide clinically useful information and serve as a promising non-invasive diagnostic and stratification tool. Furthermore, in-depth analysis of the EV molecular cargo could potentially yield higher sensitivity and specificity than sampling from whole plasma, as cancer-derived EVs are likely to be enriched in diagnostically relevant molecules, which are not detectable upon mixing with highly abundant blood proteins. Finally, extracellular vesicle content is highly stable for extended periods, due to the EV lipid bilayer that protects the cargo from degradation by extracellular proteases and other enzymes^9,10^, thereby making clinical diagnostics more robust.

In this study, we set out to explore the possibility of EV proteins to discriminate between different cancer types or even subtypes of the same cancer, which would considerably increase the potential of EVs as accessible biomarker source. Breast cancer (BC) is one of the most commonly diagnosed cancers in women with nearly 1.7 million new cases and more than half-a-million deaths occurring each year worldwide^11^. BC is highly heterogeneous and can take on distinct pathological features and clinical implications depending on the molecular subtype^12^. In routine clinical practice, breast tumors are treated differently depending on the expression levels of estrogen receptor (ER), progesterone receptor (PR), and the human epidermal growth factor receptor *HER2/ERBB2*^13^, and each subtype is associated with different treatment responses and clinical outcomes.

Amongst the well-documented BC subtypes, HER2-positive and triple-negative (defined by the absence of *ER, PR, HER2/ERBB2* expression) breast tumors present distinct challenges in diagnosis and therapeutic needs. For instance, HER2-positive tumors can be treated rather effectively with HER2-blocking antibodies if the HER2 amplification is diagnosed accurately^14^. Triple-negative breast cancer (TNBC), on the other hand, has an especially poor prognosis. These tumors are currently lacking targeted therapies and are limited only to conventional chemotherapy options, which are most effective if administered in a timely manner^15,16^. This molecular distinction makes accurate classification prior to treatment critical for positive therapeutic outcome.

Current BC diagnostic methods (mammography, breast ultrasound, and MRI) have boosted early detection, however, these tests can often be ambiguous and have documented drawbacks. For example, mammography is associated with poor detection of cancers in dense breasts, high rates of false positives leading to unnecessary biopsy testing, and over-diagnosis resulting in aggressive treatments of malignancies that may not progress during the individual’s lifetime^17,18^. As such, there is still a shortage of reliable blood-based BC biomarkers for non-invasive and robust diagnosis, classification, response prediction and prognostic monitoring of breast cancer^18,19^. In this respect, extracellular vesicles have high prospects as potential liquid biopsy^20,21^.

In breast cancer, analysis of blood-circulating extracellular vesicles holds great promise for early detection and diagnosis. A growing number of studies suggest that BC-derived EVs are enriched in various cancer-associated molecules such as oncogenic proteins (*HER2, EGFR, FAK*, survivin, *EMMPRIN, CD24*, and *EpCAM*) and miRNAs compared with healthy controls^22–26^. Over the past years, the use of mass spectrometry (MS)-based proteomics technologies enabled in-depth EV proteome profiling, which led to the discovery of an increasing number of EV proteins with altered expression in patients with BC compared with healthy controls. Recent work by Chen et al. reported more than 100 phosphoproteins to be significantly higher in BC plasma-derived EVs^10^. In addition, glypican-1 (*GPC1*), fibronectin, and the developmental endothelial locus-1 (*EDIL3*) are highly abundant in EVs isolated from the peripheral blood of BC patients compared with healthy individuals^27–30^.

However, while these studies nicely demonstrate the potential of blood-derived EVs as potential biomarker source, they did not use matched case-controls, including different cancer types or specific cancer subtypes. Therefore, currently reported BC biomarkers are mostly generic cancer markers, lacking in specificity toward breast cancer. Furthermore, to the best of our knowledge, none of these studies has reported a clear correlation between the EV-associated protein biomarkers and the molecular subtypes of breast cancer. In this study, we focus on defining EV subtype-specific signatures that could play a role in non-invasive diagnostic testing. To this end, we profiled the proteomes of extracellular vesicles secreted by (breast) cancer cell lines with a special emphasis on the TNBC and HER2 subtypes. Moreover, to further understand paracrine oncogenic signaling mediated through EVs, we performed a comprehensive EV phosphoproteome analysis, revealing differential phosphorylation status of protein kinases in EV subtypes.

Remarkably, our data revealed very distinct proteomic profiles across the different cell line-derived EVs that reflect the unique biology of its breast cancer subtype. We show here that the EV proteomes cluster based on their respective molecular subtypes where the full cellular proteomes do not enable BC subtyping. Our EV proteomes provide extensive information on subtype-specific biological processes and molecular pathways and reveal protein signatures unique to each subtype, with the distinct advantage of having positive markers for the TNBC tumors. Importantly, these in vitro molecular signatures remain valid in a full proteome context, and also specifically in serum-derived EVs of BC patients but not normal, CRC or NSCLC patients, indicating the potential of the herein presented findings for BC classification and biomarker discovery.

## Results

### Extracellular vesicles isolation and characterization

**Fig. 1:**
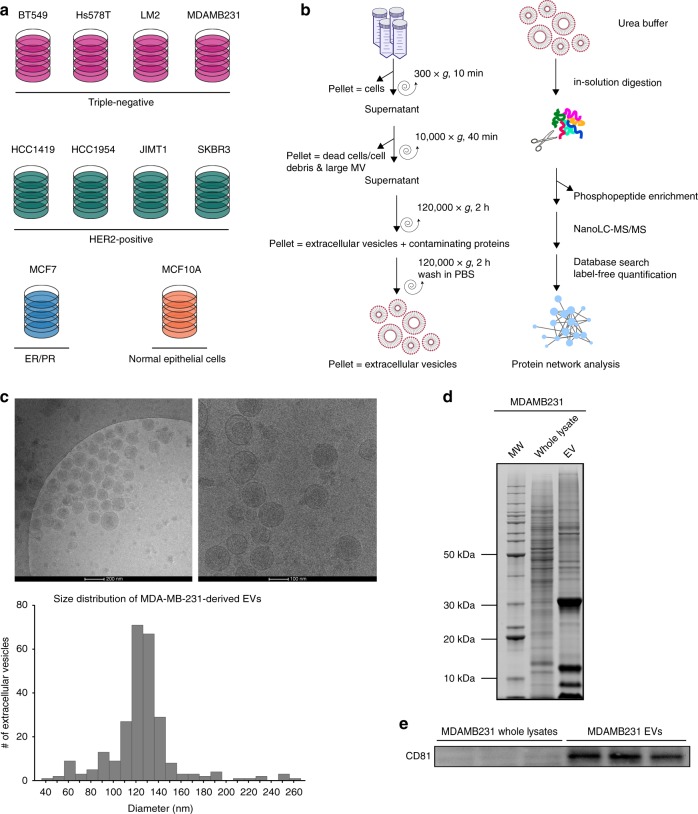
Extracellular vesicle isolation and characterization. **a** Cell lines used for EV isolation. **b** EV isolation and (phospho)proteomics workflow. **c** Cryo-EM images of purified MDAMB231 EVs and size distribution of the isolated vesicles determined using ImageJ software. **d** Comparative SDS-PAGE profile of a whole-cell lysate and an EV lysate. **e** Western blot of exosomal marker CD81 in the MDAMB231 EV lysate and whole-cell lysate (the full blot can be found in Supplementary Fig. 1)

In this study, we seek to identify protein signatures in extracellular vesicles that may be used as biomarkers for breast cancer diagnosis, subtyping, and disease monitoring. To profile the (phospho)proteomes of breast cancer-derived EVs, we isolated EVs secreted from nine different breast cancer cell lines; one low-grade luminal-type non-metastatic breast cancer cell line (MCF7), four triple negative (Hs578T, BT549, MDA-MB-231, LM2), and four HER2 positive/ER negative (HCC1954, HCC1419, JIMT1, SKBR3). In addition, EVs derived from a cell line considered as resembling normal breast epithelial cells (MCF10A) were studied as a benign control (Fig. 1a). The EV isolation workflow including the subsequent mass spectrometry analysis steps is illustrated in Fig. 1b, and further detailed in the Methods section. As shown in Fig. 1c, EVs purified from MDA-MB-231 cells were mostly in the expected diameter range of 100–140 nm, validating the high selectivity of EV preparations. Moreover, by comparing the proteins extracted from EVs against the MDA-MB-231 total cell lysate on SDS-PAGE, we observed distinct proteomic profiles between the two lysates, indicating a different EV protein composition (Fig. 1d). In-gel digestion and MS analysis of the most prominent protein bands identified members of the 14-3-3 protein family (~30 kDa), and PPIA, profilin and S100A proteins (~10 kDa), which are known to be highly enriched in EVs (within the top 100 most often published EV markers described in public EV databases). Finally, Western blot detection showed that EV lysates are highly enriched for CD81, a known marker for exosomes (Fig. 1e, Supplementary Fig. 1). Collectively, these data demonstrate a selective enrichment of EVs from cell culture conditioned media.

### Proteome profiling of extracellular vesicles

**Fig. 2:**
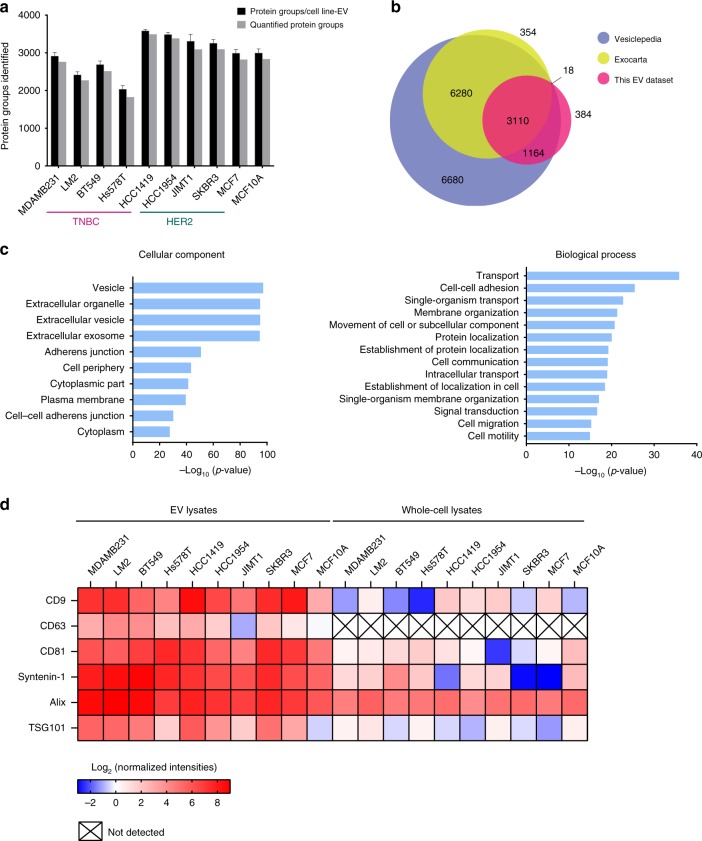
Mass spectrometry-based profiling of EV proteomes. EVs from 10 breast (cancer) cell lines (each *n* = 4 biologically independent samples) were analyzed by MS. **a** Bar plot of the total number of identified (black) and quantified (gray) proteins in EVs from each cell line. **b** Venn diagram of proteins identified in the EV samples compared with proteins annotated in the Exocarta and Vesiclepedia databases. **c** Gene ontology enrichment analysis of the EV proteins identified using the DAVID database. **d** Heatmap illustrating the enrichment of exosomal protein markers in the EVs compared with the whole-cell lysates based on their relative abundances

To compare EV protein contents across 10 breast (cancer) cell lines, isolated EVs (*n* = 4) were digested in-solution and analyzed by liquid chromatography-tandem mass spectrometry (nanoLC-MS/MS) on a high-resolution mass spectrometer (Q-Exactive HF). In total, we identified 4992 vesicular proteins (1% FDR) from all EVs isolated . Next, we applied retention time alignment and label-free quantification, and, to ensure confidence, we selected 4676 proteins that were quantified in at least three out of four biological replicates for subsequent analyses (Fig. 2a; ‘quantified protein groups’). Interestingly, despite equal injection amounts and comparable total intensities during MS analysis, the number of proteins detected in the EVs secreted from the TNBC cell lines was consistently lower compared with the rest. This was, however, not due to difference in dynamic range (Supplementary Fig. 2), suggesting TNBC-EVs comprise a less diverse protein content.

Following this initial selection step, we took this highly confident set of EV proteins and cross-referenced it with two publicly available extracellular proteome databases (‘Vesiclepedia’ and ‘Exocarta’). This analysis shows that >90% of our identifications have been previously implicated in extracellular function (Fig. 2b), and we additionally identified another 384 proteins that were not previously documented to exist in EVs.

To gain functional insight into the proteomic cargo in purified EVs, we performed gene ontology (GO) analysis using the DAVID database v6.7. For this, we obtained the whole-cell proteomes of all the 10 breast (cancer) cell lines, which we used as background to obtain GO enrichments (Fig. 2c). Proteins identified from EVs were strongly enriched in the following cellular components: vesicle (*n* = 1554, *p*-value = 6.98E−98) and extracellular vesicle (*n* = 1350, *p*-value = 1.18E−95), further confirming the efficient EV isolation. Interestingly, biological processes relating not only to transport, cell–cell adhesion and cell communication, but also signal transduction and cell migration were significantly enriched, accurately reflecting the established role of EVs in mediating breast cancer intercellular signaling (Fig. 2c)^5,25^.

Amongst the 4676 proteins quantified across all measurements, we found several proteins to be commonly identified in the EVs from all 10 cell lines, composing likely the core EV proteins needed for conserved EV function. These include known EV proteins such as 14-3-3 proteins, integrins, annexin proteins, and cytoskeletal proteins that are needed to mediate EV formation, orchestrate binding to recipient cell, and direct fusion with recipient cell plasma membrane (Supplementary Fig. 3). Within this core EV proteome, we also identified various tetraspanins (CD63, CD81, and CD9), as well as Alix (PDCD6IP) and syntenin-1 (SDCBP), which have a documented role in exosome release. The enrichment of each of these markers in the EV lysates relative to the whole-cell lysates is shown in Fig. 2d. While Alix has been used as an EV-specific marker to assess EV purity^31,32^, we detected also high levels of Alix from total cell lysates, suggesting that Alix may not be a suitable marker in the context of breast cancer. Finally, to further assess the purity of EV isolation from common co-isolated contaminants, we also evaluated the abundance of negative EV markers such as the apolipoproteins A1/2 and B (APOA1/2, APOB) and albumin (ALB) (Supplementary Fig. 4)^33^.

### EV proteomes reveal BC molecular subtype

**Fig. 3:**
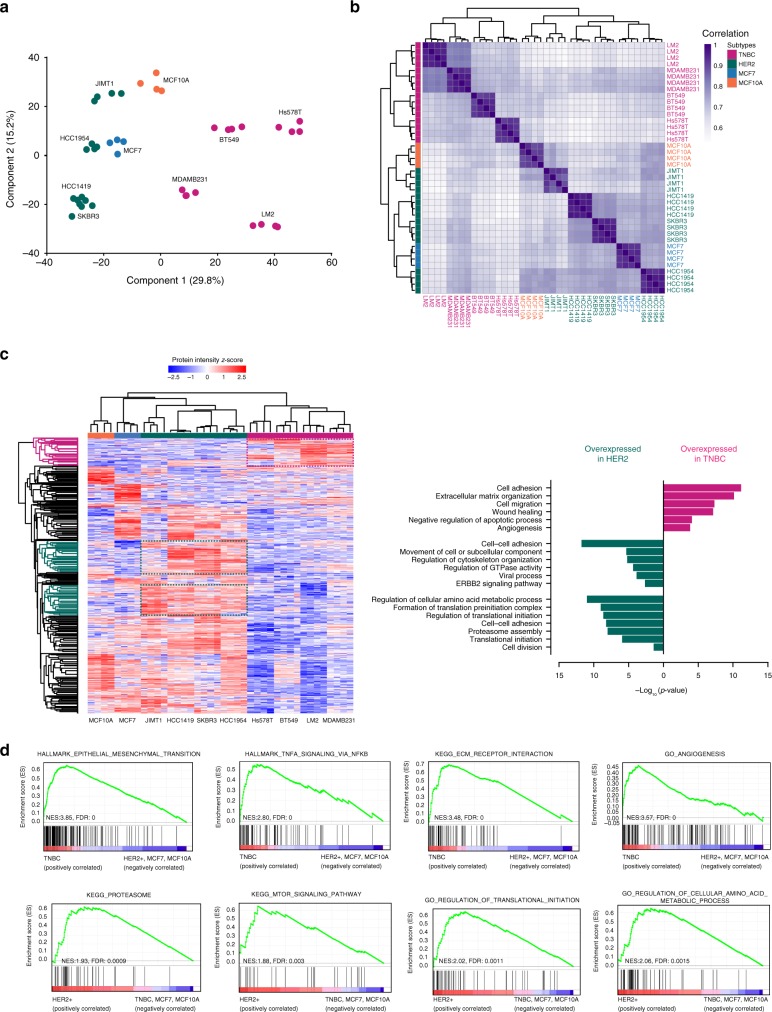
EV proteomes stratify breast cancer by molecular subtype. **a** Principal component analysis (PCA). TNBC subtype cell line clusters distinctly from HER2-positive cell lines, MCF7, and MCF10A. Within each cell line, all biological replicates (*n* = 4 biologically independent samples) cluster close to each other. **b** Hierarchical clustering of Pearson correlations. Average correlations between biological replicates was >0.9, whereas average correlations between the same subtype cell lines was >0.7. **c** Heatmap of *z*-scored protein intensities of the differentially expressed EV-proteins (ANOVA, FDR<0.05) after unsupervised hierarchical clustering, and gene ontology analysis of proteins enriched in the TNBC- and HER2-positive EVs (see Supplementary Fig. 3). **d** Top gene sets enriched in EVs of the TNBC or HER2-positive BC subtype EVs, by GSEA. Proteins in each subset of EVs are ranked by GSEA based on their differential expression level. Whether a pre-specified pathway is significantly over-represented toward the top or bottom of the ranked gene list in each subtype is evaluated using the enrichment score (green line). Black vertical lines mark positions where members of a particular pathway appear in the ranked list of genes

To assess similarities and differences in the EV profile of these 10 different EV populations on a global scale, we next performed principal component analysis (PCA). As shown in Fig. 3a, all TNBC cell lines cluster together, and segregate away from the HER2-positive cell lines. In addition, within the same cell line, all biological replicates of EV proteomes cluster tightly together, again demonstrating the high reproducibility of EV isolation and robust label-free MS quantification. In agreement with PCA, unsupervised hierarchical clustering of the Pearson correlations showed segregation of the EV proteomes into two main clusters based on the breast cancer subtypes (Fig. 3b), where the correlations between biological replicates generally exceeded 0.9. Collectively, these analyses demonstrate that proteomic cargo in EVs indeed carries significance in BC subtype stratification and that deeper elucidation of these exported proteins may aid in better understanding of BC disease etiology.

Next, we set out to define signatures presenting differential protein abundance in EVs derived from either TNBC or HER2-positive breast cancer cells. By unsupervised hierarchical clustering of 2299 ANOVA significant (FDR 5%) differential proteins (Fig. 3c), TNBC cell lines formed a distinct cluster (red) while the HER2-positive cell lines formed another major cluster (green), albeit closer to the ER/PR-positive MCF7 (blue) and benign MCF10A (orange) cell lines. Remarkably, numerous subtype-specific protein clusters that reflect BC etiology were evident from the heatmap. Gene ontology analysis within TNBC-specific protein clusters (Fig. 3c) revealed that TNBC-EVs were significantly enriched with proteins involved in metastatic processes such as extracellular matrix organization, cell migration, and angiogenesis. All these pathways recapitulate the highly invasive and metastatic nature of TNBC. On the other hand, EVs from the HER2-positive breast cancer cells were significantly enriched in proteins that function in cell–cell adhesion, translation initiation and the ERBB2 signaling pathway, which reflect the biology of HER2 tumors and their proliferative nature, as evident by the strong enrichment in terms related to increased protein synthesis, a process that has been associated with cell proliferation, a hallmark that is common to HER2-positive tumors^13,34,35^.

Gene set enrichment analysis (GSEA) further revealed that TNBC-EVs were strongly enriched in gene sets linked to aggressive signaling pathways promoting cancer cell migration, invasion and metastasis. These included the pathways epithelial-mesenchymal transition, *TNFa* signaling via *NFkB*, ECM-receptor interaction and angiogenesis (Supplementary Table 1a). HER2-positive EVs, on the other hand, showed enrichment in gene sets controlling metabolism, proteasome regulation and translation initiation (Supplementary Table 1b). The profiles of these most enriched gene sets are shown in Fig. 3d.

### EV phosphoproteome

Since phosphoproteins have recently been identified in EVs^10^, we next sought to profile our BC EVs for phosphorylated proteins, in search of phosphorylated signaling molecules that can execute or amplify signaling function directly in the recipient cell. Using a phosphopeptide enrichment strategy described previously^36^, we detected a total of 25,800 phosphosites (10,443 phosphopeptides), of which 15,264 had a localization probability over 0.75, were mapped to 2305 proteins. Of these phosphosites, 4602 could be quantified in at least three out of four replicates across all EV samples (Supplementary Fig. 5A). By comparing the current phosphoproteomics dataset against publicly available databases (Vesiclepedia, Exocarta), 300 phosphoproteins were detected for the first time, to the best of our knowledge, in EVs (Supplementary Fig. 5B). Interestingly, an over-representation of phosphorylated tyrosine residues was observed (3% vs <1% in cellular phosphoproteomes) (Supplementary Fig. 5C), in agreement with previous reports^10,37^. We found that a significant portion of the phosphorylated EV cargo originates from the plasma membrane (e.g., cell adhesion and cytoskeletal proteins as well as membrane receptors, phosphatases, etc.), alluding to the exciting possibility that EVs incorporated by recipient cells may directly impart signaling function, bypassing the need for initiating phosphorylations on membrane receptors. Potential processes that may be mediated through transfer of EV phosphoproteins include cytoskeleton organization, signal transduction, cell motility, and cell migration (Supplementary Fig. 5D).

**Fig. 4:**
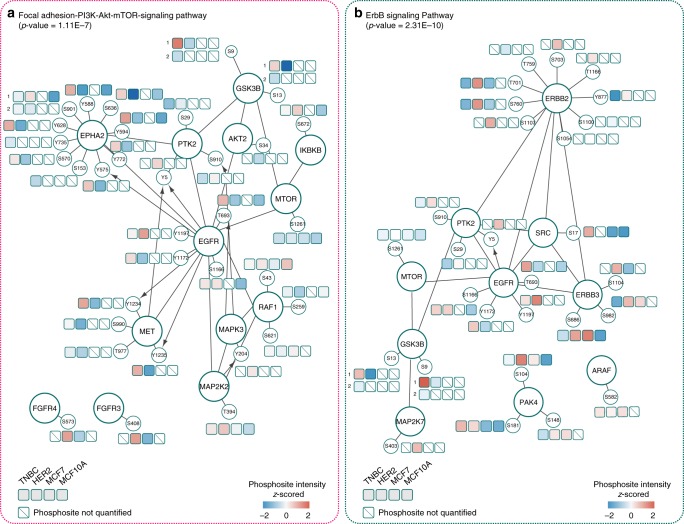
BC subtype-specific EV protein kinase networks. **a** Component of the Focal adhesion-PI3K-Akt-mTOR signaling pathway, and **b** components of the ErbB signaling pathway, visualized using PhosphoPath. Quantitative information for each EV subtype is featured in the accompanying boxes. Each box represents the median phosphosite intensity. The lines between nodes mark protein–protein interactions reported in Biogrid. Kinase-substrate interactions from PhosphositePlus are visualized by an arrow

To further understand the role of these phosphoproteins in EV uptake, release and potential intercellular signaling, we annotated the EV phosphoproteome for interaction pathways using the Reactome Cytoscape Plugin (FDR<0.001)^38^. The phosphoproteins identified feature in functional pathways including signaling by Rho GTPases, integrins and various protein kinases (*Met*, *EGFR*, *ErbB*, and EPH-Ephrin). Since activated protein kinases may continue to function and amplify phosphorylation-driven signaling upon uptake into recipient cells, we focused our analyses on the phosphorylated protein kinases detected in our dataset (101 kinases, 243 phosphosites). Three dominant classes of phosphorylated kinases were identified from EVs: (I) receptor tyrosine kinases (e.g., *ERBB2*, *EPHA2*, *AXL*, *MET*, *EGFR*); (II) non-receptor tyrosine kinases (e.g., *SRC, PTK2, YES1, LYN*); (III) MAP kinases (e.g., *ERK1* (*MAPK3*)). We visualized in PhosphoPath^39^, with quantitation, the most prominent kinase signaling pathway identified from TNBC and HER2-positive EVs. In EVs from TNBC (Fig. 4a), the active protein kinases enrich strongly for the focal adhesion-PI3K-Akt-mTOR signaling pathway (*p*-value = 2.88E−06). HER2-positive EVs, on the other hand, contain active kinases in the ErbB signaling pathway (*p*-value = 1.13E−08), as exemplified by hyperphosphorylations of *ERBB2*, *ERBB3,* and *PAK4* (Fig. 4b).

Interestingly, many of the upregulated phosphosites are critical in kinase activation and further downstream signaling. For instance, we detected *EPHA2* hyperphosphorylations (Y588/Y594; Y735) in the EVs. These are key binding sites for other downstream signaling molecules (e.g., *VAV2/3* GEFs and p85) and are critical for EPHA2-mediated angiogenesis and migration^40^. We also detected autophosphorylated *EGFR* C-terminus (Y1197, Y1172), a critical region of EGFR that induces downstream ERK signaling through recruitment of SHC and GRB2^41–43^. As another example, *ERBB2* phosphorylations in the kinase domain (Y877)^44^, in the SHC-interacting domain (Y735)^43^ and at T701, a site of feedback regulation by *ERK1/2*^45^ were detected all from EVs. These data together present exciting evidence that apical kinases in activated forms may be transferred to recipient cells to hijack signaling regulation.

Encouraged by the observation of *EPHA2*, *EGFR*, and *ERBB2* phosphorylation, we next assessed the prevalence of activated kinases in EVs. Many kinases contain a well-defined region, called the t-loop, whose phosphorylation is required for enzymatic activation^46^. We searched in the EV phosphoproteome for phosphorylated peptides that contain t-loop activating sites as a proxy for kinase activation. In addition to *EPHA2* (t-loop Y772), we also found activated *MET* (t-loop Y1234/1235), *CDK7* (t-loop T170), *CDK12* (t-loop T893), and *ERK1* (t-loop Y204) in EVs, with their phosphosites unambiguously localized. In addition, we also found the phosphorylated t-loops of numerous other kinases that share the same peptide sequence in the t-loop and were thus not distinguishable only with t-loop containing peptides. These include *DYRK2/DYRK4*, *GSK3A/GSK3B*, and *YES1/FYN/LCK/SRC*. Nonetheless, it was evident that EVs contain many activated kinases that could potentially further alter paths of phosphorylation signaling in the recipient cell.

### Subtype-specific EV protein signatures

**Fig. 5:**
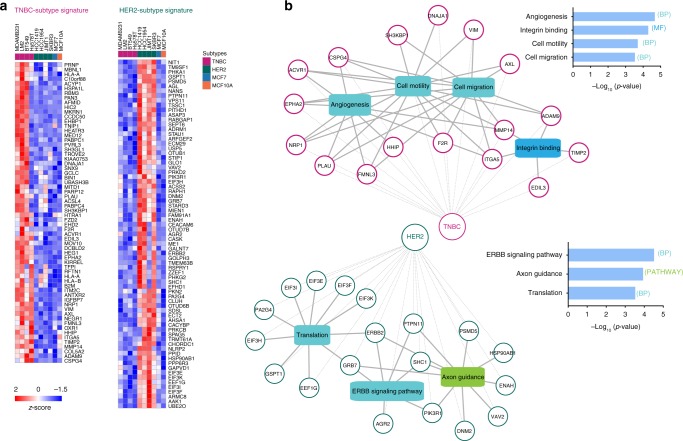
BC subtype-specific EV biomarker signatures. **a** Heatmap of subtype-specific EV protein markers (*z*-scored medians). **b** Functional enrichment analysis of the TNBC and HER2-positive EV subtype signatures using the ToppCluster tool (FDR correction, *p*-value < 0.05)^66^

Given the ability of EV contents to distinguish BC subtypes, and the observed strong gene set enrichments that reflect cancer etiology (Fig. 3), we next proceeded to extract specific signature proteins that could serve as BC subtype-specific biomarkers in circulation. To this end, we found in total 64 proteins that were significantly more abundant in TNBC-EVs, and 73 proteins that were instead more abundant in the HER2-positive EVs (Fig. 5a). The levels of these signature proteins were largely conserved across cell lines of the same BC subtype. TNBC-specific signature proteins featured prominently angiogenesis (*PLAU*, *ADAM9*, *EPHA2*), cell motility and cell migration (*VIM*, *AXL*), and integrin binding (*ITGA5*, *TIMP2*), whereas HER2-positive signature proteins functioned mainly in ERBB signaling (*GRB7*, *SHC1*), translation (EIFs) and axon guidance (*DNM2*, *PIK3R1*) (Fig. 5b).

Since strong differentiating properties emerged from EVs of different BC cell lines, and these corroborated very well with the clinical phenotype of tumors driven by these etiologies, we additionally wanted to verify the presence of the selected markers in the corresponding whole-cell proteomes. Label-free quantification resulted in 5581 protein groups being quantified across the 10 cell lines, after filtering the data for at least two valid values out of three replicates. Interestingly, both PCA analysis and hierarchical clustering of the Pearson correlations did not show a distinct clustering of the cell lines based on their respective subtype, nor did unsupervised hierarchical clustering of the significantly changing proteins (FDR < 0.05) (Supplementary Fig. 6A, B). However, when we extracted the EV signature proteins intensities from the whole-cell lysate proteomes for hierarchical clustering (67/73 of the HER2-specific markers and 47/64 of the TNBC-specific markers identified in the whole-cell lysates) we could observe segregation into the classical breast cancer subtypes (Supplementary Fig. 6C). These data provide further support that EVs are representative of tumor cells and that EV protein profiles have higher potential diagnostic power than tumor biopsies, making circulating EVs superior non-invasive diagnostic and stratification surrogates.

### Mapping of subtype-specific EV signatures in human serum EVs

To confirm the validity of our proteomic findings and strengthen their potential utility and translation toward clinical applications, we next sought to verify the trends in EV signature proteins in circulating EVs derived from a small cohort of TNBC- and HER2-positive breast cancer patient serum samples (five patients per subtype). Moreover, to assess the specificity of our signature proteins to breast cancer we included in our comparative analysis serum-derived EVs from five healthy donors, five colorectal cancer (CRC), and five lung cancer patients (NSCLC) as well.

**Fig. 6:**
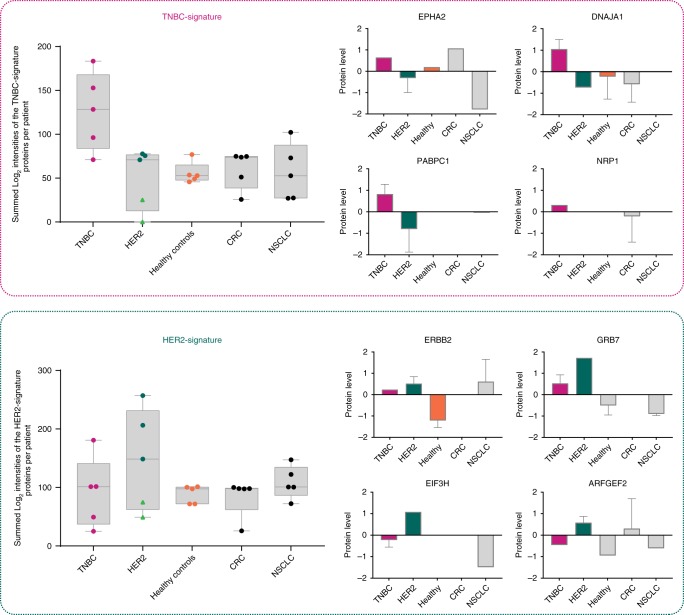
Mapping of the EV subtype-specific signature proteins to human serum-derived EVs. Summed intensities of a subpanel of the TNBC- and HER2-signature proteins identified per patient-derived EVs (*n* = 5 biologically independent samples). Comparison of protein expression levels in each cancer type and healthy controls are given for four selected proteins per subtype-signature proteins (*z*-scored normalized Log_2_ intensities). Light green triangles indicate low EV protein yield

The EV enrichment was performed similarly by differential ultracentrifugation, however, the EV yield was not always high in every sample (Supplementary Fig. 7). This, we believe, is largely due to long-term storage of the frozen serum samples that were not intended initially for this study, and/or due to sample handling (e.g., additional freeze-thaw), which could have impacted EV stability and isolation. Despite this, a subpanel of the BC subtype-specific EV markers could still be confidently validated in patient-derived EVs (Supplementary Table 3). More importantly, as it can be seen from Fig. 6, the summed intensities of the TNBC- and HER2-signature proteins detected per patient sample, showed prominent specificity of the EV-protein signatures for the respective breast cancer subtypes. Although, in the case of the HER2-signature the protein markers were poorly detected in two out of five HER2 patient samples, where general EV protein yield was low (light green triangles). Some representative TNBC-signature proteins included *EPHA2*, *DNAJA1*, *PABPC1*, and *NRP1*, which showed higher expression levels in the patient TNBC-EVs compared with the HER2-positive EVs. Similarly, *ERBB2*, *GRB7*, *EIF3H* and *ARFGEF2* were amongst the most discriminative protein markers for the HER2-positive patient serum-derived EVs. We envision that EV isolation from fresh plasma in future experiments could only further improve the detection sensitivity, and hence subtype-specific BC diagnostic power.

Altogether, these findings indicate that breast cancer subtyping using extracellular vesicles is feasible and that our proteomic data can serve as the basis to develop clinical diagnostics toward enabling better BC therapeutic decisions.

## Discussion

In recent years, EVs have gathered interest as potential non-invasive liquid biopsies^47^. This is largely due to the realization that EVs are capable of transferring disease related signaling molecules, and the increased capabilities of detecting such disease-associated molecules present at extremely low amounts, and from highly complex backgrounds such as patient plasma or serum^9^. Here, we explored the potential of using EVs as circulating biomarkers for the detection and classification of breast cancer by comparing extensively the EV cargo proteins, phosphoproteins, and protein kinases from 10 breast (cancer) cell lines. Unsupervised hierarchical clustering of differential protein expression between TNBC and HER2 cell lines revealed numerous subtype-specific protein clusters that reflect BC etiology. TNBC-EVs were significantly enriched with proteins involved in metastatic processes while EVs from the HER2-positive BC subtype were significantly enriched in proteins that function in cell proliferation. Moreover, our phosphoproteomics data revealed the exciting possibility that EVs may directly transfer activated signaling proteins, bypassing the need to be first phosphorylated in the recipient cell prior to downstream signaling. We postulate that phosphoproteins transferred to recipient cells may be directly functional; with some of these phosphorylated proteins being hyperphosphorylated receptors (e.g., *ERBB2* and *3*, *EGFR*) or activated kinases to begin with (e.g., *MET*, *ERK1*, *GSK3*). In this respect, our phosphoproteomics dataset is one of few available resources that can be further explored for candidates that regulate paracrine signaling, which plays a vital role in colonizing distant sites. In line with this hypothesis, a number of recent studies have showed direct transfer of activated receptors and subsequent downstream signaling activation in the recipient cells^7,48,49^.

To achieve the goal of subtype-specific BC detection, we selected from our BC subtype-specific EV proteomes, 64 TNBC, and 73 HER2-positive EV proteins, which can be used as potential signatures for diagnostic and/or stratification between different breast cancer subtypes, illustrating disease etiology considerably better than whole-cell proteome signatures. These markers are not only indicative of BC subtypes, but also seemingly reflect the well-documented differential invasive or proliferative nature of TNBC and HER2-positive breast cancers, respectively^50–52^. Part of the marker panels we report here overlap with a comprehensive proteomics study of 40 breast tumors published previously^53^. Tyanova et al. showed that HER2-positive breast tumors also express high intracellular *ERBB2* and *GRB7*. This provides cross-verification that high EV *ERBB2* and *GRB7* might have originated from intracellular overexpression of these proteins. In addition, four TNBC markers (*AGR2*, *HID1*, *MLPH*, *STMN1*) we found regulated in EVs were also in agreement with the trends reported by Tyanova at al. We were intrigued that, other than these six markers, no additional overlapping trends were found with the rest of their 19-signature proteins, hinting at selective packaging of tumor proteins into EV cargo and in line with our in vitro data on whole-cell proteomes. This observation further highlights that EV profiling can open up a fresh avenue in BC diagnosis, with the potential to uncover biomarkers that will not be identified even in extensive tissue cancer proteomics.

The subtype-specific BC biomarker signatures we had identified existed not only in EVs from cells cultured in vitro, but also in HER2-positive and TNBC patient serum samples. Although serum sample collection and storage were not optimized for EV collection, this lowered the EV protein recovery somewhat for some patients, we could still confidently identify numerous BC subtype-signature proteins from our panel. Even more promising was the observation that summing the intensities of all TNBC- and HER2-signature proteins detected per patient sample could further improve the specificity of EV-protein signatures for the respective breast cancer subtypes. We believe the strength of the signatures is partly derived from the fact that these were obtained in a relatively clean environment (cultured cell lines), preventing ‘contamination’ by other  cell types. This resulted in a truly BC subtype-specific EV protein signature, which we could partly confirm in real-life patient samples. Moreover, the multi-protein character of the signatures is eminently suited to deal with patient heterogeneity, where EV protein composition and abundances will be highly variable, resulting in high variability of their detection. As shown here, the summing of those signature proteins that were observed in the highly variable patient-specific background, does show enrichment in their respective BC subtype.

The data presented herein thus provides a proof-of-concept of the utility of EV biomarkers in BC subtyping. With the data presented in this work, the goal is next to validate the trends we observed in larger cohorts of (fresh) plasma-derived EVs, to establish the associated diagnostic and clinical value. As pointed out by recent studies aiming to identify liquid-biopsy signatures in other cancers, such as prostate and sarcoma, the biggest challenge in implementing the identified biomarker signatures in clinical use lies in their further validation and verification in large prospective studies^54,55^. Moreover, we envision the molecular subtyping of EVs presented here as a promising option, equally applicable in other cancer sub-classifications, which may form the bases for future studies. Our current study is thus the first step, which we believe provides convincing evidence that analysis of the EV-proteomic cargo and the associated molecular pathways have strong potential for identifying circulating biomarker signatures for diagnosis and management of diseases such as cancer.

## Methods

### Cell lines and cell culture

MDAMB231, BT549, Hs578T, MCF7, SKBR3, HCC1419, HCC1954 (obtained from the ATCC), LM2, and JIMT1 (provided by The Netherlands Cancer Institute, NKI) were cultured in DMEM supplemented with 10% FBS (v/v), 100 U mL^−1^ penicillin, 100 µg mL^−1^ streptomycin, and 100 µg mL^−1^ L-Glutamine. MCF10A cells (ATCC) were grown in DMEM/F12 supplemented with 5% (v/v) horse serum, 20 ng mL^−1^ EGF, 0.5 mg mL^−1^ hydrocortisone, 10 μg mL^−1^ insulin, and 100 ng mL^−1^ cholera toxin. All cells were maintained in a humidified incubator at 37 °C with 5% CO_2_. All cell lines have been tested for mycoplasma contamination.

### Serum samples collection

Frozen serum samples from female patients diagnosed with triple-negative breast cancer, HER2-positive breast cancer, colorectal cancer, and non-small-cell lung carcinoma were obtained from the general clinical laboratory at the Netherlands Cancer Institute, after approval by the local Institutional Review Board. Plasma samples from healthy volunteers were obtained after informed consent and in accordance to the ethics board of Sanquin. For each cancer type and healthy controls we received frozen serum samples from five individuals. All breast cancer patients were diagnosed with stage 3 or 4 breast cancer. All colorectal and lung cancer patients were stage 4. Inclusion criteria for healthy female control donors were a negative medical history for any malignant disease and a minimum of 40 years of age. All blood samples were taken before treatment, except for one HER2-positive breast cancer patient who received PTC-pertuzumab treatment prior to blood collection.

### Extracellular vesicle isolation from cell lines

**Cell lines**. Bovine EV-depleted media was obtained by overnight ultracentrifugation at 100,000 × *g*, at 4 °C, in a medium supplemented with 20% FBS (in a Sorvall T-865 rotor). For EV isolation, cells were left in culture until they had reached 70–80% confluency, washed three times with PBS, and further cultured in EV-depleted medium (10% EV-depleted FBS final) for 48 h before collection of the conditioned medium for EV purification. EVs were isolated by differential ultracentrifugation as previously described by Thery et al. with some modifications^56^. Briefly, conditioned medium (120 mL; 6 × 15-cm dishes per replicate) was centrifuged at 300 × *g* for 10 min to pellet cells. Then, the supernatant was centrifuged for 40 min at 10,000 × *g* in a Sorvall T-865 rotor to pellet apoptotic bodies, cellular debris, and large microvesicles. The collected media was ultracentrifuged at 120,000 × *g* for 2 h to pellet smaller extracellular vesicles, including exosomes. Finally, the EV pellet was resuspended in PBS, carefully washed and centrifuged at 120,000 × *g* for 2 h to collect the final EV pellets. All centrifugation steps were performed at 4 °C.

### Circulating EVs from frozen

Circulating EVs from frozen serum samples were isolated as described above. Approximately 3 mL of cell-free serum per patient were thawed on ice. Then, the serum was diluted with 17 mL PBS and was centrifuged at 10,000 × *g* for 40 min. EVs were then harvested by ultracentrifugation at 120,000 × *g* for 2 h at 4 °C. Next, the EV pellet was washed in PBS followed by a second step of ultracentrifugation at 120,000 × *g* for 2 h at 4 °C.

### Cryo-electron microscopy

For the preparation of thin vitrified specimens, a 3-μL drop of the sample was transferred to a glow discharged Quantifoil micromachined Holey Carbon (R 2/2) TEM grid (Quantifoil Micro Tools GmbH, Jena, Germany) and held by the Vitrobot mark IV tweezer (FEI, Eindhoven, The Netherlands). The temperature in the Vitrobot chamber was set at room temperature (25 °C) and the humidity to 100%. Excess of the sample was removed by blotting filter papers and the grid was immediately frozen in liquid ethane and transferred into a Tecnai20 LaB6 electron microscope (FEI, Eindhoven, The Netherlands). The specimen’s temperature was held below −165 °C during the whole procedure to prevent ice formation. An Eagle 4k × 4k CCD camera (FEI, Eindhoven, The Netherlands) was used to record micrographs of the vesicles, which was done in Tif format with a nominal under focus of 3 μm. Vesicle diameter was measured using the ImageJ software.

### Western blot analysis

EVs and cells were lysed by Urea buffer (8 M urea buffer in 50 mM ammonium bicarbonate pH 8.5, Complete mini EDTA-free protease inhibitor cocktail (Roche), PhosSTOP phosphatase inhibitor cocktail (Roche)) and protein concentration was determined using a Bradford assay (Bio-Rad). Immunoblotting was performed using a 12% Criterion XT precast gel (Bio-Rad) and a 0.45 μm nitrocellulose membrane (Bio-Rad Laboratories). The mouse monoclonal antibody CD81 (5A6, Santa Cruz Biotechnology, Santa Cruz CA) was used as a primary antibody. Protein detection was performed using ECL agent (Pierce) and a GeneGnome scanner (Syngene) for chemiluminescence imaging.

### Sample preparation for proteomics analysis

Purified EV pellets were lysed in Urea lysis buffer (8 M urea buffer in 50 mM ammonium bicarbonate pH 8.5, Complete mini EDTA-free protease inhibitor cocktail (Roche), PhosSTOP phosphatase inhibitor cocktail (Roche)) and further sonicated using a Bioruptor® Plus sonication device (Diagenode) for 15 cycles (30 s on, 30 s off). Whole-cell lysates from each cell line were also collected along with the EVs for proteome analysis. Cells were harvested, washed twice in PBS and subsequently lysed in urea buffer. Prior to in-solution digestion, the total protein concentration was quantified by Bradford assay (Bio-Rad, Hercules, CA, USA) as recommended by the manufacturer. For label-free quantification, input amounts were normalized based on the total protein contents. Subsequently, 30 and 100 μg of extracted proteins per EV and WCL sample, respectively, were used for (phospho)proteomics analysis. Proteins were then reduced (4 mM DTT) and alkylated (8 mM iodoacetamide) before being digested with LysC (Wako, Richmond, VA, USA) for 4 h (enzyme/substrate ratio 1:75) at 37 °C. Samples were diluted four times and digested further by Trypsin (Promega, Madison, WI, USA) (enzyme/substrate ratio 1:100) at 37 °C overnight. Finally, the digestion was quenched with 5% formic acid and the resulting peptides were cleaned-up on SepPak C18 cartridges (Waters Corporation, Milford, MA) and further used directly for single run proteome analysis or submitted to phosphorylation enrichment.

### Mass spectrometry analysis

For label-free quantification, 2 μg of each EV and WCL digest were analyzed by nanoLC-MS/MS on an Orbitrap Q-Exactive HF Mass Spectrometer (ThermoFisher Scientific, Bremen) coupled to an Agilent 1290 Infinity Ultra-High Pressure Liquid Chromatography (UHPLC) system (Agilent Technologies), operating in reverse phase (C18) equipped with a Reprosil pur C18 trap column (100 µm × 2 cm, 3 µm, Dr. Maisch) and a Poroshell 120 EC C18 analytical column (75 µm × 50 cm, 2.7 µm, Agilent Technologies). After trapping for 5 min in a flow rate of 0.05 ml/min in 100% solvent A (0.1% FA in H_2_O), peptides were eluted with a 160 min LC gradient from 10 to 36% solvent B (0.1% FA, 80% ACN) at a flow rate of 300 nL/min. The mass spectrometer was operated in data-dependent acquisition mode, automatically switching between MS and MS2. Full scan MS spectra were acquired using the following settings: full-scan automatic gain control (AGC) target 3e6 at 60,000 resolution; scan range 375–1600 *m*/*z*; Orbitrap full-scan maximum injection time 20 ms. HCD MS2 spectra were generated for up to 12 precursors with a normalized collision energy of 27%. The fragment ions were acquired at a resolution of 30,000 (isolation window of 1.4 *m*/*z*) with an AGC target value of 1e5 charges and a maximum injection time of 100 ms. The dynamic exclusion was set to 24 s.

### EV phosphopeptide enrichment and MS analysis

Phosphopeptide enrichment was performed using a combination of Fe(III)-IMAC cartridges and an automated setup, the AssayMAP Bravo Platform (Agilent Technologies) as described previously^36^. Briefly, Fe(III)-NTA cartridges were primed with 250 μL of 0.1% TFA in ACN and equilibrated with 250 μL of loading buffer (80% ACN/0.1% TFA). EV samples containing 25 μg total protein were dissolved in 200 μL of loading buffer and loaded onto the cartridge. The columns were washed with 250 μL of loading buffer, and the phosphorylated peptides were eluted with 25 μL of 1% ammonia directly into 25 μL of 10% formic acid. Subsequently, the samples were dried down in a vacuum centrifuge. Next, phosphopeptides were reconstituted in loading buffer containing 20 mM citric acid and 1% formic acid and analyzed by nanoLC-MS/MS on a Q-Exactive HF (ThermoFisher Scientific, Bremen) coupled to an Agilent 1290 Infinity System (Agilent Technologies). As previously described, eluted phosphopeptides (amount corresponding to enrichment of 25 μg of total EV digest) were delivered to a trap column (100 μm i.d. × 2 cm, packed with 3 μm C18 resin, Reprosil PUR AQ, Dr. Maisch, Ammerbuch, Germany) at a flow rate of 5 μL/minute in 100% loading solvent A (0.1% FA, in HPLC grade water). After 5 min of loading and washing, peptides were transferred to an analytical column (75 µm i.d. × 50 cm, packed with 2.7 µm Poroshell 120 EC C18, Agilent Technologies) and eluted at room temperature using an 70 min with an LC gradient from 8 to 32% solvent B (0.1% FA, 80% ACN). The Q-Exactive HF was operated in data-dependent acquisition mode using the following settings: full-scan automatic gain control (AGC) target 3e6 at 60,000 resolution; scan range 375–1600 *m*/*z*; Orbitrap full-scan maximum injection time 20 ms; MS2 scan AGC target 1e5 at 30,000 resolution; maximum injection time 100 ms; normalized collision energy 27; dynamic exclusion time 12 s; isolation window 1.4 *m*/*z*; 12 MS2 scans per full scan.

### Data processing

All raw MS files were searched with the MaxQuant software (versions 1.5.8.3 and 1.6.2.3)^57^. MS/MS spectra were searched by Andromeda against a reviewed *homo sapiens* database (UniProt, March 2017, 20,168 entries) using the following parameters: trypsin digestion; maximum of two missed cleavages; cysteine carbamidomethylation as fixed modification; oxidized methionine, protein N-terminal acetylation, and serine/threonine/tyrosine phosphorylation (for the phosphoproteome data analysis only) as variable modifications. Mass tolerance was set to 4.5 and 20 ppm for the MS1 and MS2, respectively. The protein and PSM False Discovery Rate (FDR) were set to 1%. Peptide identifications by MS/MS were transferred between runs to replace missing values for quantification, with a 0.7-min window after retention time alignment.

### Data analysis, statistics and reproducibility

All data were analyzed using the Perseus software^58^, Microsoft Excel and R statistical software. Raw intensities extracted by MaxQuant were log2 transformed, and normalized by median of replicates for label-free quantification in proteomics and phosphoproteomics. Missing values were replaced by imputation according to normal distribution with a downshift of 1.8 SDs and a width of 0.3 SDs. Only class I phosphorylation sites (localization probability *p* > 0.75) were used in subsequent phosphoproteome analyses. For hierarchical clustering, normalized intensities were first *z*-scored and then clustered using Euclidean as a distance measure for column and row clustering.

Vesiclepedia (Version 3.1, 2017) and Exocarta (July 2015) were downloaded to map previously reported EV proteins^59–61^. Gene ontology (GO) analyses were performed with Database for Annotation, Visualization and Integrated Discovery (DAVID) v6.8, using all the proteins identified by the whole-cell lysate proteomics experiment as background^62,63^. Gene set enrichment analysis (GSEA) was performed using Broad GSEA version 3.0, using gene set collections from the Molecular Signatures database (MSigDB) v6.1^64,65^. Interaction and pathway enrichment of phosphorylated kinases were performed using Cytoscape software platform (version 3.6.1) with the PhosphoPath plug-in^39^, with imported data sources from PhosphositePlus for kinase-substrate interactions, BIOGRID for protein–protein interactions, and WikiPathways for pathway information. The full proteomes were used similarly as a background for enrichment.

To assess the reproducibility of the experiments within the biological replicates of each cell line-derived EVs (*n* = 4) we employed principal component analysis (PCA), which was performed using the Perseus’ built-in tool. Moreover, we also examined the Pearson correlation coefficients, which between the biological replicates exceeded 0.9. To identify the differentially expressed proteins across the EV subtypes, we performed an ANOVA test followed by a Benjamini-Hochberg multiple testing correction with a 5% FDR. Amongst them, the EV subtype-signature proteins were selected by extracting in Perseus the proteins with the most distinct expression profiles, between the different subtypes.

### Reporting summary

Further information on research design is available in the Nature Research Reporting Summary linked to this article.

## Supplementary information

Supplementary Information

Description of Additional Supplementary Items

Supplementary Data 1

Supplementary Data 2

Supplementary Data 3

Reporting Summary

## Data Availability

All mass spectrometry proteomics data have been deposited to the ProteomeXchange Consortium via the PRIDE partner repository with the dataset identifier PXD012162. All quantified proteins (EVs and whole-cell lysate) and phosphosites are made available as supplementary datasets 1–3. All other data and materials are available from the corresponding author.
