# Supplementary material for: Proteomic profiling of extracellular vesicles allows for human breast cancer subtyping
Source: Commun Biol. 2019 Sep 3;2:325. doi: 10.1038/s42003-019-0570-8 (PMC6722120; doi:10.1038/s42003-019-0570-8)
Supplement: Supplementary file 1 — Supplementary Information [file 42003_2019_570_MOESM1_ESM.pdf]

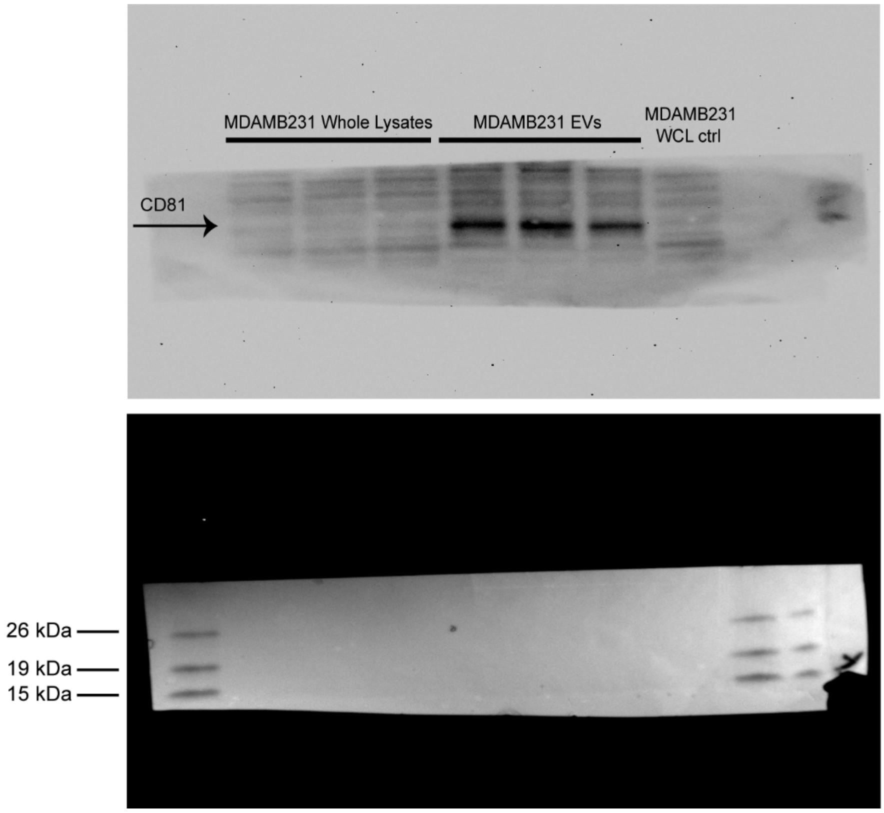

Supplementary Figure 1. CD81 expression in MDAMB231 whole cell lysate and EV lysates. Uncropped blot image.

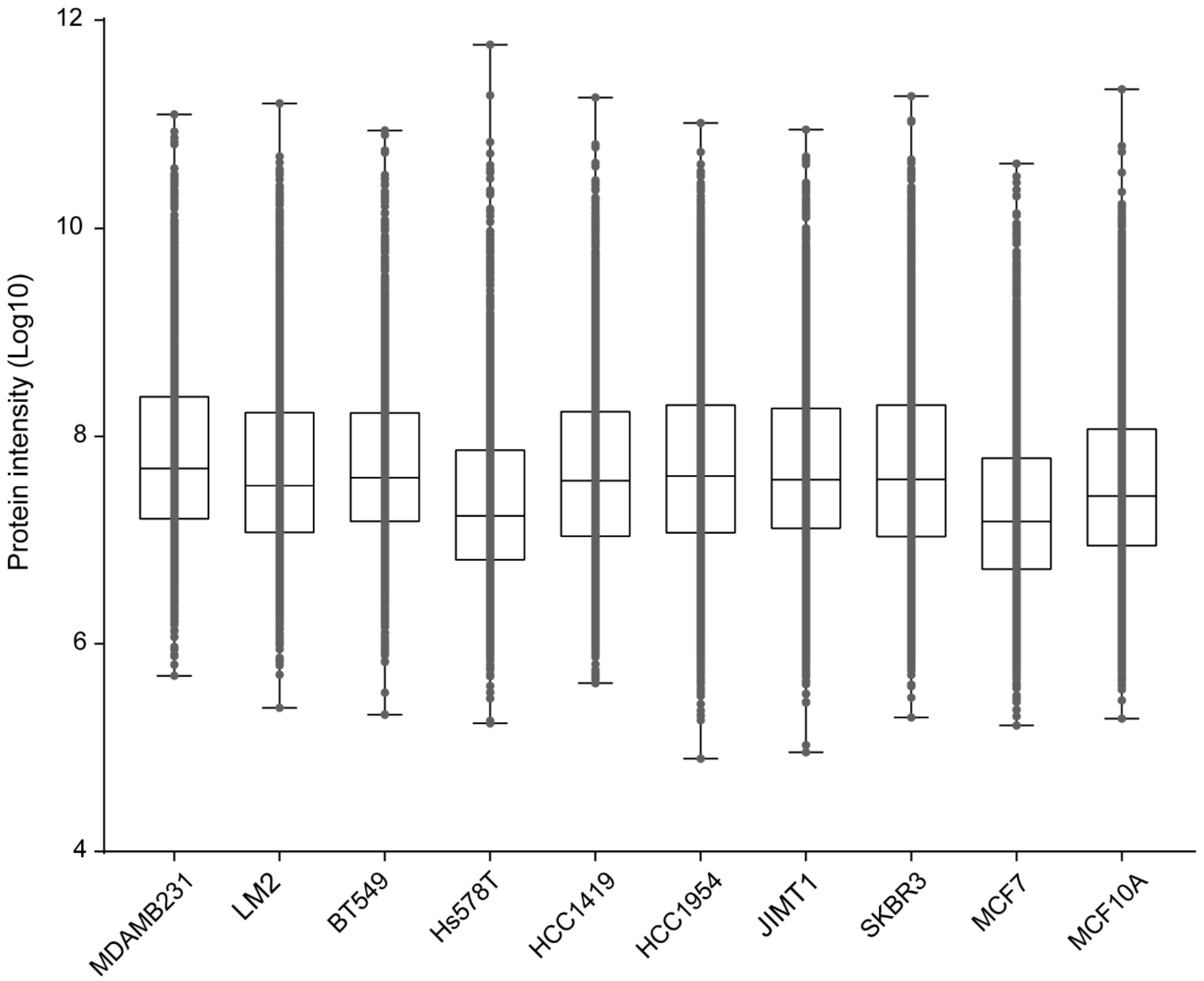

Supplementary Figure 2. EV proteins intensities. Boxplots illustrating the differences in protein abundances between all EV proteins per cell-line derived EVs.

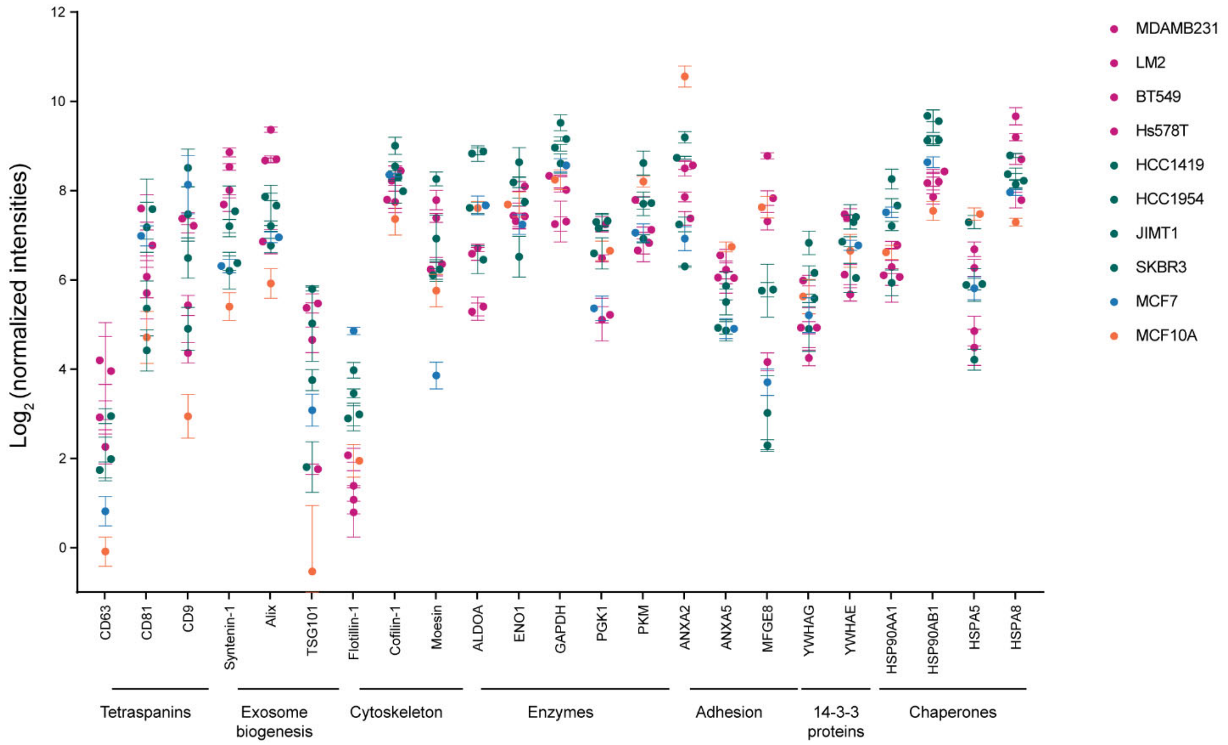

Supplementary Figure 3. Common EV markers identified across all EV samples. Enrichment of common EV protein markers measured across all EVs.

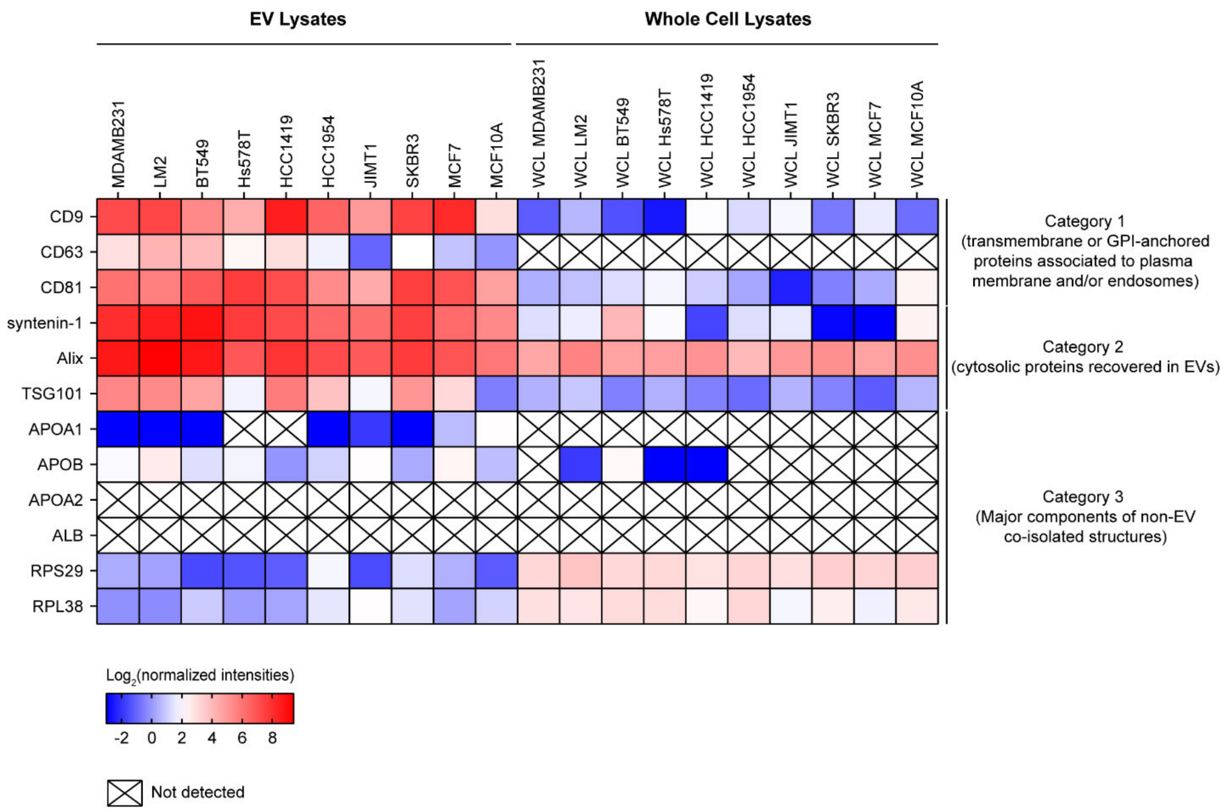

Supplementary Figure 4. Protein content-based EV characterization according to the recommendations of the recently published MISEV2018 guidelines [1]. Heatmap displaying the abundance of at least one of each of the three categories of protein markers detected in the EV preparations compared to the whole cell lysates.

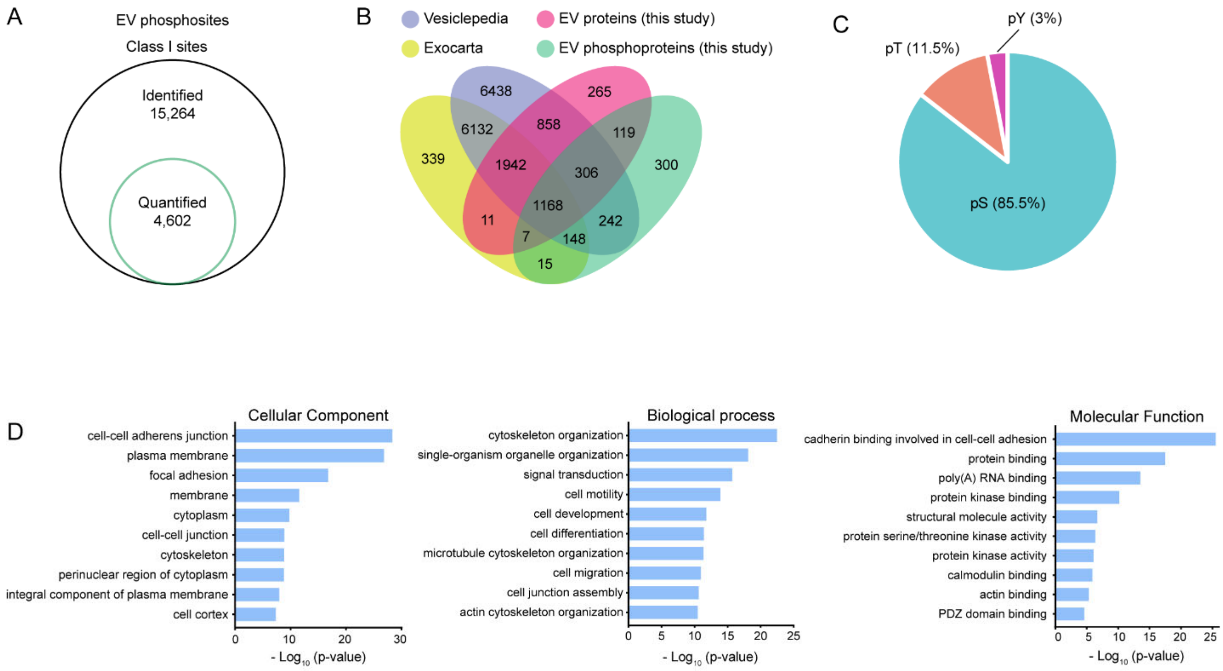

Supplementary Figure 5. EV Phosphoproteome. A. All phosphosites identified and quantified with a localization probability >0.75. **B.** Overlap between the EV phosphorylated proteins, EV proteins and proteins annotated in the Exocarta and Vesiclepedia databases. **C.** Distribution of identified phosphosites containing serine (pS), threonine (pT) and tyrosine (pY). **D.** Gene ontology enrichment analysis of the EV phosphoproteins. Only top 10 enriched terms (lowest p-values) are shown in each ontology.

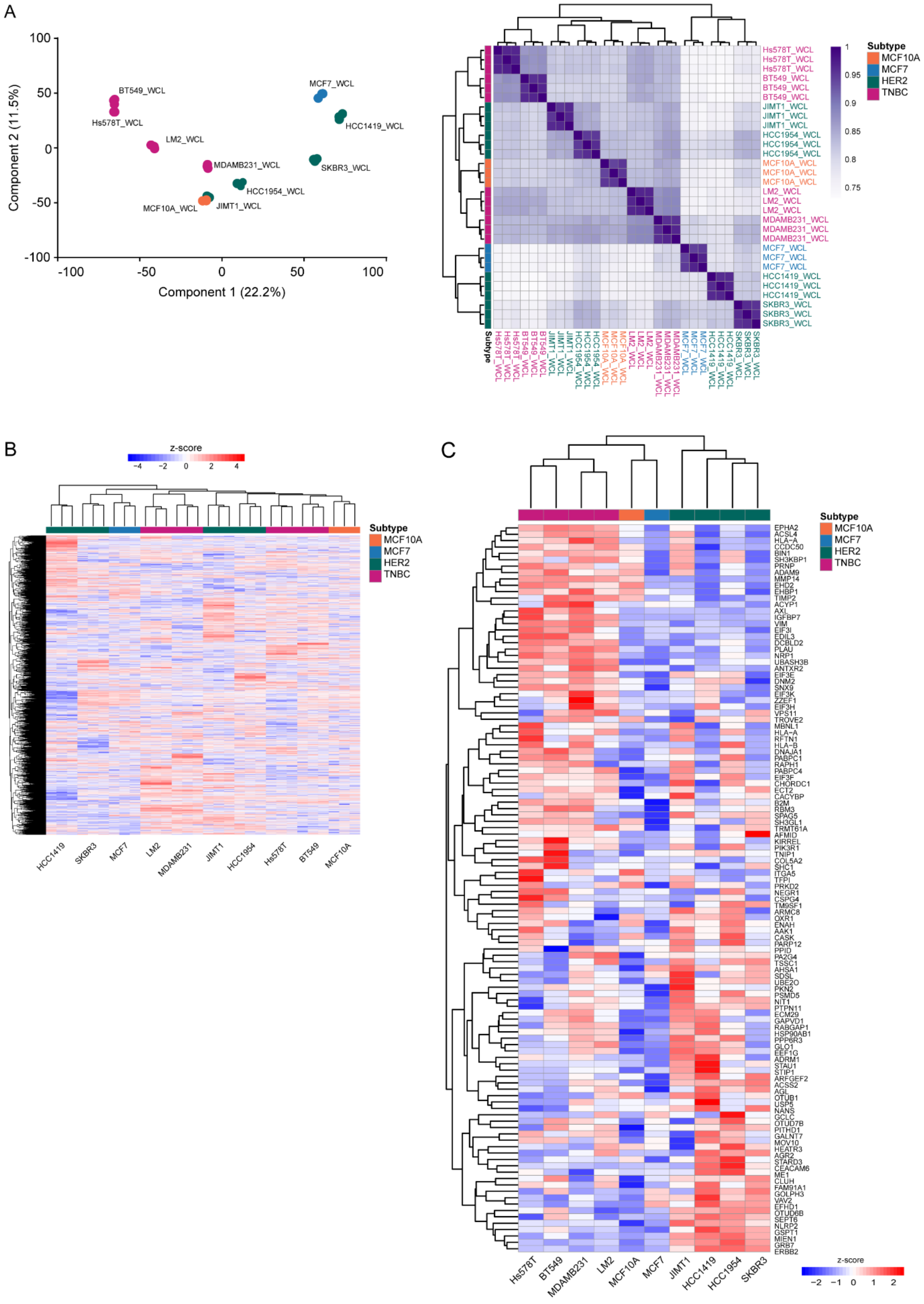
A
Component 1 (22.2%)
Component 2 (11.5%)
BT549_WCL
Hs578T_WCL
LM2_WCL
MDAMB231_WCL
MCF10A_WCL
JIMT1_WCL
HCC1954_WCL
MCF7_WCL
HCC1419_WCL
SKBR3_WCL
Subtype
MCF10A
MCF7
HER2
TNBC
B
z-score
HCC1419
SKBR3
MCF7
LM2
MDAMB231
JIMT1
HCC1954
Hs578T
BT549
MCF10A
C
Hs578T
BT549
MDAMB231
LM2
MCF10A
MCF7
JIMT1
HCC1419
HCC1954
SKBR3
z-score

Supplementary Figure 6. Proteomic profiling of whole cell lysates. A. Principal component analysis (PCA) and hierarchical clustering (Pearson correlation) based on protein abundance. **B**. Heatmap of z-scored protein intensities of the differentially expressed proteins (ANOVA, FDR<0.05) **C.** Heatmap of the protein abundances (z-scored medians) of the TNBC and HER2-positive EV subtype-specific signatures as detected in the whole lysates of the different cell lines.

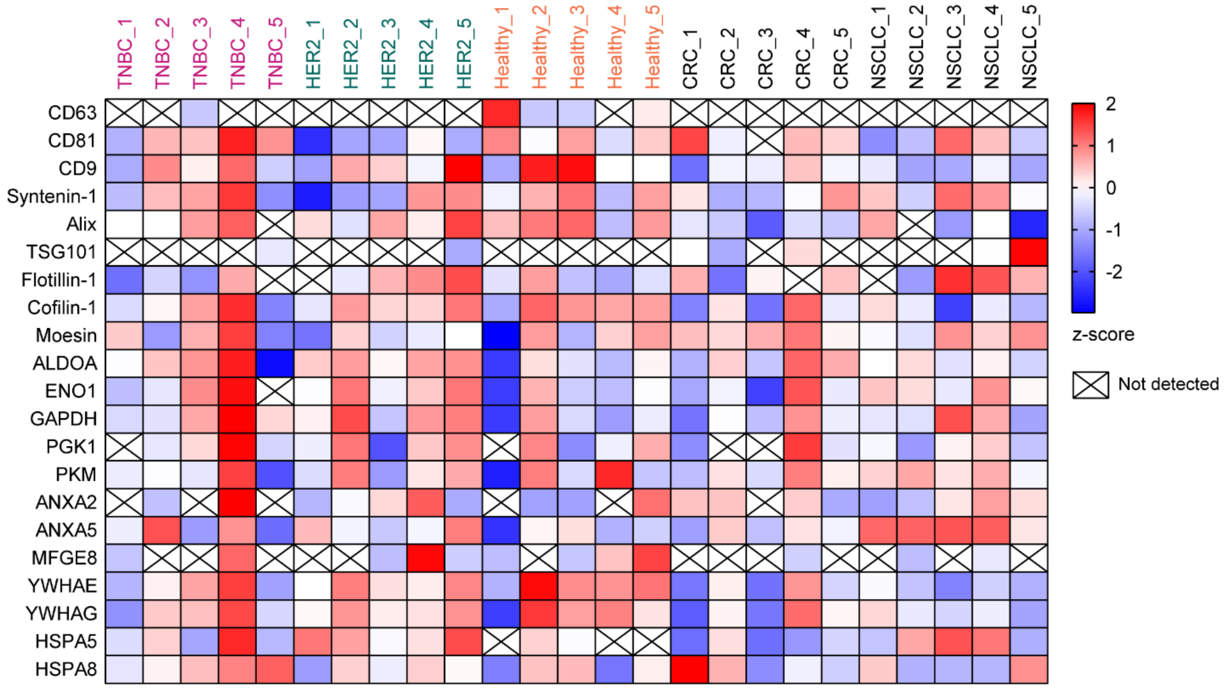

Supplementary Figure 7. EV markers in the serum-derived EVs. Comparison of the most common EV markers identified across the different patient-derived EVs.

Supplementary Table 1a. GSEA of EV proteins associated with the TNBC subtype.

NAME	SIZE	ES	NES	NOM p-val	FDR q-val (cutoff<0.05)
HALLMARK_EPITHELIAL_MESENCHYMAL_TRANSITION	133	0,6540719	3,85466	0	0
HALLMARK_TNFA_SIGNALING_VIA_NFKB	56	0,5475942	2,8069596	0	0
HALLMARK_INFLAMMATORY_RESPONSE	57	0,5104872	2,5062516	0	0
HALLMARK_ANGIOGENESIS	17	0,6461675	2,3825557	0	0
HALLMARK_IL6_JAK_STAT3_SIGNALING	20	0,5345839	2,137414	0	0
HALLMARK_UV_RESPONSE_DN	72	0,36810255	2,0255773	0	0
HALLMARK_COAGULATION	67	0,35265657	1,8565571	0	0,002917834
HALLMARK_KRAS_SIGNALING_UP	53	0,3424629	1,7302884	0	0,006893382
HALLMARK_IL2_STAT5_SIGNALING	76	0,3367854	1,682648	0	0,010212418
HALLMARK_MYOGENESIS	75	0,29578114	1,6497003	0	0,014501635
HALLMARK_KRAS_SIGNALING_DN	24	0,36971372	1,5274572	0,05940594	0,032568324
HALLMARK_APICAL_SURFACE	19	0,39960963	1,5025907	0,05	0,03325844
KEGG_ECM_RECEPTOR_INTERACTION	58	0,68450683	3,4805853	0	0
KEGG_CYTOKINE_CYTOKINE_RECEPTOR_INTERACTION	36	0,63154024	2,742483	0	0
KEGG_HEMATOPOIETIC_CELL_LINEAGE	18	0,6910933	2,6168797	0	0
KEGG_CELL_ADHESION_MOLECULES_CAMS	52	0,4960803	2,4235094	0	0
KEGG_HEDGEHOG_SIGNALING_PATHWAY	16	0,632326	2,2780492	0	0
KEGG_HYPERTROPHIC_CARDIOMYOPATHY_HCM	39	0,43377253	1,9352672	0	0,004275001
KEGG_DILATED_CARDIOMYOPATHY	40	0,41028127	1,9226185	0	0,004182468
KEGG_COMPLEMENT_AND_COAGULATION_CASCADES	23	0,43467057	1,774263	0	0,013973859
KEGG_FOCAL_ADHESION	123	0,2921633	1,7647603	0	0,013210429
KEGG_AXON_GUIDANCE	68	0,30095914	1,5747403	0	0,04199096
GO_BLOOD_VESSEL_MORPHOGENESIS	132	0,4749768	3,0534868	0	0
GO_EXTRACELLULAR_STRUCTURE_ORGANIZATION	155	0,51751465	3,0283368	0	0
GO_MULTICELLULAR_ORGANISM_METABOLIC_PROCESS	35	0,5923268	2,7073529	0	0
GO_BRANCHING_MORPHOGENESIS_OF_AN_EPITHELIAL_TUBE	46	0,5344634	2,6892874	0	0
GO_MULTIVESICULAR_BODY_ORGANIZATION	27	0,62724566	2,651198	0	0
GO_MULTI_ORGANISM_ORGANELLE_ORGANIZATION	22	0,6650792	2,642555	0	0
GO_MULTICELLULAR_ORGANISMAL_MACROMOLECULE_METABOLIC_PROCESS	34	0,5981032	2,6414802	0	0
GO_ANGIOGENESIS	112	0,46464446	2,5782516	0	0

GO_MULTI_ORGANISM_MEMBRANE_ORGANIZATION	26	0,62258494	2,5722544	0	0
GO_COLLAGEN_FIBRIL_ORGANIZATION	24	0,6237296	2,5593307	0	0
GO_EXOCRINE_SYSTEM_DEVELOPMENT	16	0,662179	2,5359578	0	0
GO_SPROUTING_ANGIOGENESIS	17	0,6563773	2,4822295	0	3,03E-04
GO_POSITIVE_REGULATION_OF_OSTEOBLAST_DIFFERENTIATION	21	0,6507444	2,483986	0	3,23E-04
GO_VASCULOGENESIS	18	0,682346	2,49471	0	3,46E-04
GO_TISSUE_REMODELING	35	0,55216223	2,4950726	0	3,73E-04
GO_POSITIVE_REGULATION_OF_OSSIFICATION	29	0,60827136	2,5107129	0	4,04E-04
GO_ARTERY_MORPHOGENESIS	18	0,66412765	2,4665298	0	5,34E-04
GO_MORPHOGENESIS_OF_A_BRANCHING_STRUCTURE	56	0,47195292	2,4819207	0	5,65E-04
GO_VASCULATURE_DEVELOPMENT	165	0,41238368	2,4274163	0	0,00117318
GO_TISSUE_MORPHOGENESIS	183	0,32239702	2,3943727	0	0,001523794
GO_NEGATIVE_REGULATION_OF_CYTOKINE_PRODUCTION	58	0,44710025	2,358784	0	0,002016261
GO_ENDODERMAL_CELL_DIFFERENTIATION	21	0,5644342	2,2764976	0,009259259	0,003029655
GO_FORMATION_OF_PRIMARY_GERM_LAYER	49	0,4784209	2,2600026	0	0,003274219
GO_CARTILAGE_DEVELOPMENT	35	0,5093435	2,241029	0	0,003535487
GO_CELL_SEPARATION_AFTER_CYTOKINESIS	16	0,64576644	2,2345152	0	0,003921339
GO_SUBSTRATE_ADHESION_DEPENDENT_CELL_SPREADING	25	0,53278077	2,2163155	0	0,004617054
GO_CONNECTIVE_TISSUE_DEVELOPMENT	50	0,44570002	2,222353	0	0,004622033
GO_HETEROPHILIC_CELL_CELL_ADHESION_VIA_PLASMA_MEMBRANE_CELL_ADHESION_MOLECULES	16	0,62963915	2,1985648	0	0,00490929
GO_REGULATION_OF_OSSIFICATION	49	0,45304665	2,1914806	0	0,005038133
GO_CHONDROCYTE_DIFFERENTIATION	16	0,6082823	2,1852598	0	0,005704458
GO_ENDODERM_FORMATION	23	0,54011697	2,1721396	0	0,006135693
GO_ENDODERM_DEVELOPMENT	33	0,47536287	2,1616013	0	0,006641376
GO_CARDIAC_SEPTUM_DEVELOPMENT	24	0,51715547	2,1560419	0	0,006687572
GO_INTEGRIN_MEDIATED_SIGNALING_PATHWAY	53	0,40288806	2,143148	0	0,007242638
GO_EYE_MORPHOGENESIS	36	0,4561761	2,1320772	0	0,007893788
GO_REGULATION_OF_BIOMINERAL_TISSUE_DEVELOPMENT	22	0,54851544	2,105997	0	0,008175036
GO_FOREBRAIN_CELL_MIGRATION	23	0,53290296	2,1115015	0	0,008283093
GO_CARDIAC_CHAMBER_MORPHOGENESIS	26	0,50573015	2,096423	0	0,008503365
GO_POSITIVE_REGULATION_OF_SMALL_GTPASE_MEDIATED_SIGNAL_TRANSDUCTION	19	0,56095976	2,1163259	0	0,008513179
GO_EXTRACELLULAR_MATRIX_DISASSEMBLY	36	0,47907078	2,0686362	0	0,009945151
GO_REGULATION_OF_INTERFERON_GAMMA_PRODUCTION	17	0,548228	2,0525174	0	0,011888543
GO_NEGATIVE_REGULATION_OF_BMP_SIGNALING_PATHWAY	15	0,55521375	2,052625	0,006024096	0,012178508

GO_VENTRICULAR_SEPTUM_DEVELOPMENT	15	0,57592624	2,033159	0	0,012420778
GO_SKELETAL_SYSTEM_MORPHOGENESIS	50	0,41100687	2,0339324	0	0,012582812
GO_REGULATION_OF_NEURON_DIFFERENTIATION	184	0,30735973	2,0277557	0	0,01272953
GO_CARDIAC_CHAMBER_DEVELOPMENT	40	0,4031798	2,0360866	0	0,012742041
GO_ENDOTHELIAL_CELL_DIFFERENTIATION	38	0,4311473	2,0344603	0	0,012856351
GO_ARTERY_DEVELOPMENT	28	0,49421722	2,0366902	0	0,013031633
GO_GASTRULATION	62	0,39745998	2,0218575	0	0,013088268
GO_CELL_FATE_COMMITMENT	34	0,439164	2,0369241	0,011235955	0,013334693
GO_REGULATION_OF_PATHWAY_RESTRICTED_SMAD_PROTEIN_PHOSPHORYLATION	26	0,484446	2,008837	0	0,014279375
GO_EMBRYONIC_CRANIAL_SKELETON_MORPHOGENESIS	15	0,5740053	1,9799829	0,00591716	0,016566362
GO_VIRION_ASSEMBLY	31	0,46352884	1,9749091	0	0,016763268
GO_NEURAL_CREST_CELL_MIGRATION	15	0,5325115	1,975111	0	0,016907355
GO_POSITIVE_REGULATION_OF_AXON_EXTENSION	18	0,5147161	1,9716411	0,007246377	0,016953152
GO_CARDIAC_VENTRICLE_DEVELOPMENT	27	0,46217382	1,9645522	0	0,01727359
GO_SKELETAL_SYSTEM_DEVELOPMENT	120	0,35246718	1,9583784	0	0,017609386
GO_LENS_DEVELOPMENT_IN_CAMERA_TYPE_EYE	22	0,52501196	1,9570183	0	0,017674407
GO_REGULATION_OF_EPITHELIAL_CELL_MIGRATION	70	0,35567284	1,9516323	0	0,018269025
GO_REGULATION_OF_OSTEOBLAST_DIFFERENTIATION	34	0,44161913	1,9523704	0	0,018352933
GO_VASCULAR_PROCESS_IN_CIRCULATORY_SYSTEM	46	0,3952751	1,9369646	0	0,019040536
GO_DEVELOPMENTAL_GROWTH	110	0,30247438	1,9387968	0	0,019216577
GO_CELL_SUBSTRATE_ADHESION	83	0,3712283	1,934201	0	0,019352075
GO_ENDOTHELIUM_DEVELOPMENT	46	0,40493783	1,930801	0	0,019742817
GO_ORGAN_MORPHOGENESIS	229	0,295131	1,9265401	0	0,019984988
GO_HEART_MORPHOGENESIS	56	0,41074994	1,9233264	0	0,020214641
GO_DEVELOPMENTAL_GROWTH_INVOLVED_IN_MORPHOGENESIS	42	0,40527952	1,9171137	0	0,020365592
GO_REGULATION_OF_CYTOKINE_PRODUCTION_INVOLVED_IN_IMMUNE_RESPONSE	23	0,4537234	1,9174014	0	0,020669557
GO_REGULATION_OF_BMP_SIGNALING_PATHWAY	27	0,45164397	1,9122537	0	0,021088649
GO_CARDIAC_MUSCLE_TISSUE_DEVELOPMENT	41	0,4172347	1,9056252	0	0,021103678
GO_REGULATION_OF_CELLULAR_RESPONSE_TO_GROWTH_FACTOR_STIMULUS	84	0,34836173	1,9056629	0	0,021345494
GO_SEMAPHORIN_PLEXIN_SIGNALING_PATHWAY	19	0,5130929	1,894141	0,007936508	0,021367747
GO_REGULATION_OF_TRANSMEMBRANE_RECEPTOR_PROTEIN_SERINE_THREONINE_KINASE_SIGNALING_PATHWAY	76	0,36296502	1,8923814	0	0,021436442
GO_POSITIVE_REGULATION_OF_PATHWAY_RESTRICTED_SMAD_PROTEIN_PHOSPHORYLATION	22	0,48464292	1,8963794	0,007936508	0,02147812
GO_CRANIAL_SKELETAL_SYSTEM_DEVELOPMENT	17	0,5284534	1,9020303	0,007142857	0,021485602
GO_MESODERM_MORPHOGENESIS	29	0,46256396	1,8977747	0	0,021505445

GO_TUBE_MORPHOGENESIS	105	0,355346	1,9057034	0	0,021588374
GO_POSITIVE_REGULATION_OF_NEURON_DIFFERENTIATION	99	0,3515843	1,8980005	0	0,021796059
GO_POSITIVE_REGULATION_OF_EPITHELIAL_CELL_MIGRATION	43	0,40869358	1,8791727	0	0,022793131
GO_AMINOGLYCAN_BIOSYNTHETIC_PROCESS	31	0,4376072	1,8751099	0,013157895	0,023205558
GO_NEGATIVE_REGULATION_OF_SMALL_GTPASE_MEDIATED_SIGNAL_TRANSDUCTION	18	0,50944227	1,8731558	0	0,023284312
GO_BONE_MORPHOGENESIS	19	0,49676108	1,8710419	0,006802721	0,02330387
GO_EMBRYONIC_SKELETAL_SYSTEM_MORPHOGENESIS	22	0,4743787	1,8680375	0	0,023505922
GO_POSITIVE_REGULATION_OF_CELL_DEVELOPMENT	156	0,29687214	1,8608824	0	0,024234653
GO_EYE_DEVELOPMENT	93	0,32584113	1,863473	0	0,024324903
GO_POSITIVE_REGULATION_OF_SMOOTH_MUSCLE_CELL_MIGRATION	15	0,53083307	1,8579521	0,006756757	0,024560856
GO_REGULATION_OF_AXON_GUIDANCE	16	0,52336305	1,8537909	0	0,025046702
GO_REGULATION_OF_VASOCONSTRICTION	16	0,49390244	1,8509748	0,021428572	0,025570145
GO_POSITIVE_REGULATION_OF_PEPTIDYL_TYROSINE_PHOSPHORYLATION	47	0,395815	1,8493499	0	0,025582233
GO_GLAND_MORPHOGENESIS	41	0,38839436	1,8421752	0	0,026498444
GO_ENDOTHELIAL_CELL_MIGRATION	27	0,43486574	1,8311411	0,009174312	0,026813734
GO_DEVELOPMENTAL_CELL_GROWTH	32	0,4221701	1,8288609	0	0,026862297
GO_POSITIVE_REGULATION_OF_AXONOGENESIS	30	0,4234353	1,8329262	0	0,02687297
GO_REGULATION_OF_CELL_ADHESION_MEDIATED_BY_INTEGRIN	20	0,48865306	1,834026	0	0,026973195
GO_NEURAL_CREST_CELL_DIFFERENTIATION	23	0,44258225	1,8358388	0,008849558	0,026981045
GO_POSITIVE_REGULATION_OF_RESPONSE_TO_WOUNDING	34	0,4088752	1,8230587	0	0,027250363
GO_REGULATION_OF_LEUKOCYTE_APOPTOTIC_PROCESS	25	0,45156035	1,8233553	0,01923077	0,02739927
GO_REGULATION_OF_CARDIAC_MUSCLE_TISSUE_DEVELOPMENT	17	0,5020205	1,8185846	0,007246377	0,02773774
GO_POSITIVE_REGULATION_OF_LYMPHOCYTE_DIFFERENTIATION	20	0,48061365	1,8102537	0,008474576	0,029000327
GO_CAMERA_TYPE_EYE_MORPHOGENESIS	28	0,43277907	1,8011658	0	0,029057924
GO_EMBRYONIC_ORGAN_DEVELOPMENT	99	0,33302614	1,807578	0	0,029098589
GO_POSITIVE_REGULATION_OF_ADAPTIVE_IMMUNE_RESPONSE	20	0,4760458	1,8020257	0,007936508	0,02912987
GO_REGULATION_OF_NEURON_PROJECTION_DEVELOPMENT	157	0,30769774	1,7927284	0	0,029210052
GO_SMAD_PROTEIN_SIGNAL_TRANSDUCTION	20	0,46221605	1,7993208	0	0,029211683
GO_MESENCHYMAL_CELL_DIFFERENTIATION	43	0,36668444	1,8022667	0	0,02924067
GO_NEGATIVE_REGULATION_OF_CYTOKINE_SECRETION	15	0,51615787	1,7940462	0,006329114	0,029398143
GO_AXON_EXTENSION	18	0,500325	1,8026762	0,00862069	0,02944632
GO_NEGATIVE_REGULATION_OF_NERVOUS_SYSTEM_DEVELOPMENT	87	0,33975178	1,7969854	0	0,029456303
GO_MOVEMENT_IN_ENVIRONMENT_OF_OTHER_ORGANISM_INVOLVED_IN_SYMBIOTIC_INTERACTION	53	0,35942292	1,8036844	0	0,029565819
GO_POSITIVE_REGULATION_OF_PRODUCTION_OF_MOLECULAR_MEDIATOR_OF_IMMUNE_RESPONSE	19	0,48128766	1,7941167	0,007751938	0,029625054

GO_REGULATION_OF_EXTRINSIC_APOPTOTIC_SIGNALING_PATHWAY	65	0,3370369	1,7859701	0	0,02966904
GO_ORGAN_GROWTH	15	0,5196824	1,7866691	0,007246377	0,029861068
GO_CELL_MATRIX_ADHESION	61	0,35076648	1,7806145	0	0,030271184
GO_REGULATION_OF_CELLULAR_RESPONSE_TO_TRANSFORMING_GROWTH_FACTOR_BETA_STIMULUS	40	0,37921208	1,781406	0	0,030378135
GO_RESPONSE_TO_MECHANICAL_STIMULUS	65	0,34307963	1,7783157	0	0,030485334
GO_GRANULOCYTE_MIGRATION	16	0,49145204	1,7819988	0,012903226	0,030565925
GO_TUBE_DEVELOPMENT	183	0,2718963	1,7769675	0	0,030648641
GO_GLIAL_CELL_MIGRATION	19	0,5030886	1,7662284	0,015748031	0,03219082
GO_POSITIVE_REGULATION_OF_NEURON_PROJECTION_DEVELOPMENT	91	0,3497157	1,7687044	0	0,03219396
GO_NEGATIVE_REGULATION_OF_RESPONSE_TO_CYTOKINE_STIMULUS	15	0,50689816	1,7668531	0,012903226	0,032218214
GO_PATTERN_SPECIFICATION_PROCESS	75	0,3404747	1,7614292	0	0,032930486
GO_RESPONSE_TO_TRANSFORMING_GROWTH_FACTOR_BETA	63	0,3455403	1,7602364	0	0,032940038
GO_NEURON_PROJECTION_EXTENSION	25	0,44608828	1,756777	0,010526316	0,033366963
GO_NEURON_MIGRATION	33	0,39508873	1,7547618	0	0,033616193
GO_NEGATIVE_REGULATION_OF_NEURON_DIFFERENTIATION	65	0,36493754	1,7499985	0	0,03447974
GO_TAXIS	151	0,29789746	1,7436026	0	0,035418764
GO_CEREBRAL_CORTEX_DEVELOPMENT	40	0,35888454	1,7414166	0,01754386	0,0356777
GO_EMBRYONIC_SKELETAL_SYSTEM_DEVELOPMENT	29	0,4015737	1,7318122	0	0,0366899
GO_REGULATION_OF_COAGULATION	36	0,39152828	1,7327425	0	0,036874816
GO_NEGATIVE_REGULATION_OF_CELLULAR_RESPONSE_TO_GROWTH_FACTOR_STIMULUS	52	0,33384714	1,7187158	0	0,040121127
GO_CELL_CELL_ADHESION_VIA_PLASMA_MEMBRANE_ADHESION_MOLECULES	57	0,33394924	1,7145991	0	0,040719513
GO_AMINOGLYCAN_CATABOLIC_PROCESS	24	0,41859517	1,7149304	0	0,04098805
GO_EMBRYONIC_ORGAN_MORPHOGENESIS	64	0,35104787	1,7096045	0	0,04117971
GO_MESENCHYME_DEVELOPMENT	55	0,35275102	1,7109884	0	0,041202597
GO_RESPONSE_TO_OXYGEN_LEVELS	111	0,27736694	1,7031478	0	0,042961754
GO_TELENCEPHALON_DEVELOPMENT	73	0,30854952	1,6984441	0	0,043842174
GO_CELL_RECOGNITION	39	0,36040285	1,691827	0	0,044478707
GO_REGULATION_OF_RESPONSE_TO_CYTOKINE_STIMULUS	50	0,33914018	1,6921713	0	0,044716846
GO_NEGATIVE_REGULATION_OF_CELL_DEVELOPMENT	101	0,31139967	1,6925831	0	0,044887584
GO_CEREBRAL_CORTEX_CELL_MIGRATION	17	0,47708827	1,6818987	0,014492754	0,046057012
GO_CARDIAC_MUSCLE_CELL_DIFFERENTIATION	23	0,41165754	1,6831218	0,016949153	0,046110537
GO_REGULATION_OF_PEPTIDYL_TYROSINE_PHOSPHORYLATION	68	0,30306798	1,685333	0	0,046143707
GO_NEGATIVE_REGULATION_OF_TRANSMEMBRANE_RECEPTOR_PROTEIN_SERINE_THREONINE_KINASE_SIGNALING_PATHWAY	43	0,34716094	1,6770866	0,02	0,046254415
GO_LEUKOCYTE_DIFFERENTIATION	70	0,32009467	1,6755494	0	0,046319

GO_POSITIVE_REGULATION_OF_LOCOMOTION	169	0,28631788	1,6805739	0	0,04632646
GO_REGULATION_OF_MAPK_CASCADE	220	0,24141857	1,6837081	0	0,04634834
GO_REGULATION_OF_ERK1_AND_ERK2_CASCADE	69	0,3238348	1,6774836	0	0,046513483
GO_NEGATIVE_REGULATION_OF_CELL_DIFFERENTIATION	153	0,27044898	1,6775863	0	0,046808083
GO_SENSORY_ORGAN_MORPHOGENESIS	62	0,3327704	1,6707563	0	0,04732897
GO_NEGATIVE_REGULATION_OF_DEVELOPMENTAL_PROCESS	209	0,24543591	1,6714435	0	0,047469974
GO_NEGATIVE_REGULATION_OF_CELL_PROJECTION_ORGANIZATION	60	0,32950357	1,6610754	0	0,04892702
GO_POSITIVE_REGULATION_OF_NERVOUS_SYSTEM_DEVELOPMENT	134	0,30929932	1,6616591	0	0,049103152
GO_NEGATIVE_REGULATION_OF_CHEMOTAXIS	15	0,46363083	1,6640562	0,013245033	0,049116466
GO_ODONTOGENESIS	33	0,37186462	1,663018	0,011494253	0,049146734
GO_REGULATION_OF_CELL_GROWTH	144	0,2819293	1,6621399	0	0,04922694

Supplementary Table 1b. GSEA of EV proteins associated with the HER2-positive subtype.

NAME	SIZE	ES	NES	NOM p-val	FDR q-val (cutoff<0.05)
KEGG_PROTEASOME	39	0,6328617	1,9376595	0	9,29E-04
KEGG_MTOR_SIGNALING_PATHWAY	27	0,646495	1,8876325	0	0,003312365
KEGG_AMINOACYL_TRNA_BIOSYNTHESIS	25	0,6369281	1,8271948	0,001101322	0,007869756
KEGG_AMINO_SUGAR_AND_NUCLEOTIDE_SUGAR_METABOLISM	30	0,584942	1,7579488	0	0,017485362
KEGG_INSULIN_SIGNALING_PATHWAY	65	0,5273585	1,7205372	0	0,024213506
GO_REGULATION_OF_CELLULAR_AMINO_ACID_METABOLIC_PROCESS	46	0,6477915	2,0642884	0	0,001574048
GO_REGULATION_OF_TRANSLATIONAL_INITIATION	49	0,6429345	2,0295384	0	0,001181092
GO_FORMATION_OF_TRANSLATION_PREINITIATION_COMPLEX	19	0,7525628	2,013563	0	0,002104858
GO_REGULATION_OF_CELLULAR_AMINE_METABOLIC_PROCESS	53	0,624812	2,012556	0	0,001578644
GO_NIK_NF_KAPPAB_SIGNALING	60	0,6080574	1,9672834	0	0,003318744
GO_PROTEIN_MODIFICATION_BY_SMALL_PROTEIN_REMOVAL	41	0,6203065	1,925892	0	0,007109539
GO_REGULATION_OF_CELLULAR_KETONE_METABOLIC_PROCESS	71	0,5924128	1,9201525	0	0,006660051
GO_ANAPHASE_PROMOTING_COMPLEX_DEPENDENT_CATABOLIC_PROCESS	57	0,59065825	1,8951299	0	0,009691925
GO_FC_EPSILON_RECEPTOR_SIGNALING_PATHWAY	79	0,5595973	1,8696026	0	0,01265652
GO_NEGATIVE_REGULATION_OF_PROTEIN_MODIFICATION_BY_SMALL_PROTEIN_CONJUGATION_OR_REMOVAL	84	0,5556396	1,8676054	0	0,011708366
GO_AMINO_ACID_ACTIVATION	32	0,6164461	1,8605653	0	0,011940118
GO_ANTIGEN_PROCESSING_AND_PRESENTATION_OF_EXOGENOUS_PEPTIDE_ANTIGEN_VIA_MHC_CLASS_I	50	0,5830832	1,8561842	0	0,011669364
GO_REGULATION_OF_PROTEIN_UBIQUITINATION_INVOLVED_IN_UBIQUITIN_DEPENDENT_PROTEIN_CATABOLIC_PROCESS	69	0,56845564	1,8507189	0	0,012417492
GO_REGULATION_OF_INTRACELLULAR_STEROID_HORMONE_RECEPTOR_SIGNALING_PATHWAY	23	0,66132444	1,8434689	0,001109878	0,013284902
GO_FC_RECEPTOR_SIGNALING_PATHWAY	116	0,5300428	1,8343831	0	0,015038286
GO_INNATE_IMMUNE_RESPONSE_ACTIVATING_CELL_SURFACE_RECEPTOR_SIGNALING_PATHWAY	69	0,5627703	1,8341705	0	0,014098394
GO_REGULATION_OF_RNA_STABILITY	92	0,53563136	1,8127203	0	0,019877614
GO_POSITIVE_REGULATION_OF_LIGASE_ACTIVITY	67	0,5499852	1,7896364	0	0,028276332
GO_ANTIGEN_RECEPTOR_MEDIATED_SIGNALING_PATHWAY	84	0,5305962	1,7798052	0	0,031033333
GO_TRNA_METABOLIC_PROCESS	62	0,5498893	1,7770387	0	0,030905416
GO_T_CELL_RECEPTOR_SIGNALING_PATHWAY	78	0,5321811	1,7763419	0	0,029659087
GO_MYELOID_CELL_DEVELOPMENT	16	0,67675817	1,7735121	0	0,029426103
GO_TUMOR_NECROSIS_FACTOR_MEDIATED_SIGNALING_PATHWAY	61	0,54488856	1,7715914	0	0,028870353
GO_NEGATIVE_REGULATION_OF_CANONICAL_WNT_SIGNALING_PATHWAY	72	0,5332362	1,7702878	0	0,028228415
GO_ANTIGEN_PROCESSING_AND_PRESENTATION_OF_PEPTIDE_ANTIGEN_VIA_MHC_CLASS_I	64	0,5447548	1,7674999	0	0,02823807
GO_PROTEIN_POLYUBIQUITINATION	104	0,51345074	1,7649174	0	0,028155642
GO_REGULATION_OF_CELL_CYCLE_PHASE_TRANSITION	139	0,5093335	1,764354	0	0,027317923
GO_TRANSCRIPTION_COUPLED_NUCLEOTIDE_EXCISION_REPAIR	40	0,5740359	1,738614	0	0,039114345
GO_ANTIGEN_PROCESSING_AND_PRESENTATION_OF_PEPTIDE_ANTIGEN	106	0,51183224	1,7276947	0	0,044369012
GO_REGULATION_OF_LIGASE_ACTIVITY	76	0,51939005	1,7243586	0	0,044761814
GO_IMMUNE_RESPONSE_REGULATING_CELL_SURFACE_RECEPTOR_SIGNALING_PATHWAY	136	0,50059575	1,7218251	0	0,045181643
GO_POSITIVE_REGULATION_OF_PROTEIN_MODIFICATION_BY_SMALL_PROTEIN_CONJUGATION_OR_REMOVAL	101	0,5027726	1,7185997	0	0,04611859
GO_NEGATIVE_REGULATION_OF_GTPASE_ACTIVITY	20	0,6261651	1,7166989	0,001103753	0,045800176

Supplementary Table 2. Reactome Pathway analysis of the phosphorylated EV proteins.

GeneSet	Ratio Of Protein In GeneSet	Number Of Protein In GeneSet	Protein From Network	P-value	FDR	Nodes
Signaling by Rho GTPases(R)	0,0304	330	106	1,11E-16	1,05E-13	NUP107,ITSN1,PREX1,BCR,MYL6,CDCA8,IQGAP1,SFN,ARHGAP1,GMIP,INCENP,CENPA,FLNA,CENPF,TAOK1,PKN2,PKN1,CASC5,ARPC5,ITGB1,PPP1R12A,SRC,SRF,BAIAP2,PRC1,CDC20,HIST2H3A,DSN1,RACGAP1,PTK2,GRB2,CALM1,KIF14,WASL,AKAP13,CTTN,ARHGEF2,ARHGEF7,PAK2,MYO9B,ERCC6L,ACTG1,ARHGEF26,RHPN2,ECT2,YWHAB,YWHAG,ARHGEF12,ARHGEF17,ARHGEF16,CYFIP1,TRIO,DEPDC1B,NUP98,ABR,STARD13,H3F3A,NUF2,NUDC,CIT,FGD1,FGD3,KTN1,ABI1,PPP1CB,CDH1,CLIP1,OBSCN,KLC1,KLC2,SRGAP2,SRGAP1,NF2,BRK1,EVL,SOS1,HIST1H4A,RPS27,VAV2,RHOV,CTNNA1,RANBP2,TRIP10,CTNNB1,ARHGAP39,ARHGAP35,ARHGDIA,RALBP1,ARHGAP17,ARHGAP12,ARHGAP21,MYH14,MYH10,ARHGAP32,KIF18A,DIAPH3,DLG4,CKAP5,MYH9,GNA13,CLASP1,CLASP2,MAPK3,FMNL3,FMNL1,FMNL2
Processing of Capped Intron-Containing Pre-mRNA(R)	0,0215	234	73	7,77E-16	3,68E-13	NUP107,DDX39A,ZC3H11A,SF1,SRRT,SRSF10,SRSF11,CASC3,SRSF2,SRSF3,SRSF5,SNRNP200,SRSF6,SRSF7,CTNNBL1,TFIP11,PRCC,SRSF1,U2AF2,HNRNPA1,HNRNPA0,HNRNPA3,EIF4A3,CD2BP2,PCBP1,PCBP2,SYMPK,SNW1,PRPF40A,SLU7,RAE1,HNRNPA2B1,NUP155,NUP153,DDX42,RBM17,NCBP1,TRA2B,HNRNPU,NUP214,NUP210,NUP98,NUP88,HNRNPM,HNRNPK,HNRNPF,HNRNPD,HNRNPC,NDC1,SF3B2,SF3B1,THOC2,THOC5,THOC7,SNRNP27,SNRPC,CHERP,PTBP1,PPIL1,SRRM2,SRRM1,CSTF3,RNPS1,HNRNPH1,RANBP2,CWC25,CWC22,PRPF4,PRPF3,HSPA8,CPSF7,YBX1,USP39
Endocytosis(K)	0,0239	260	76	5,44E-15	1,71E-12	TFRC,PIP5K1A,EPS15L1,EGFR,AP2M1,IST1,ACAP2,ARPC5,ARRB1,VPS37D,ARFGAP2,MET,SRC,RAB11FIP1,RAB11FIP5,IQSEC1,AP2A1,WASL,GIT1,EPS15,SH3GL1,TSG101,CAV2,CAV1,IGF2R,ASAP1,ASAP2,MVB12B,MVB12A,ARPC5L,VPS35,BIN1,PARD3,VPS28,NEDD4L,EHD1,EHD2,RET,CLTC,CLTA,LDLRAP1,WWP1,HSPA1B,USP8,F2R,RAB31,RAB35,RAB5C,DAB2,PDCD6IP,VPS4B,VPS4A,PML,RAB10,HLA-C,HLA-A,HLA-E,SH3KBP1,CHMP1A,RAB8A,CXCR4,GBF1,CAPZA1,CAPZA2,CHMP4C,CHMP4B,HSPA8,CHMP2B,CHMP2A,FGFR4,FGFR3,TGFBR2,RABEP1,CHMP3,CHMP5,ERBB3
Regulation of actin cytoskeleton(K)	0,0197	214	67	1,05E-14	2,49E-12	ARAF,IQGAP1,PIP5K1A,FN1,RRAS2,EGFR,SLC9A1,ARPC5,ITGB1,ITGB5,ITGB4,ITGB3,ITGAV,PPP1R12A,ITGA4,ITGA3,ITGA2,ITGA6,ITGA5,ITGA9,SRC,BAIAP2,CRKL,ACTN4,PTK2,WASL,GIT1,ARHGEF7,PAK6,PAK2,PAK4,ACTG1,RAF1,VCL,MSN,SSH3,SSH1,ARHGEF12,CYFIP1,ARPC5L,BRAF,TMSB4X,FGD1,FGD3,ABI2,PPP1CB,CFL2,CFL1,MAP2K2,F2R,BRK1,SOS1,APC,EZR,GNG12,VAV2,ARHGAP35,PXN,MYH14,MYH10,DIAPH3,FGFR4,FGFR3,BDKRB2,MYH9,GNA13,MAPK3
Signalling by NGF(R)	0,0387	421	101	4,14E-14	7,83E-12	ITSN1,ARAF,PSMD8,PREX1,PSMD2,PSMD1,FRS2,PRKAR1B,PRKAR1A,PSME4,PRKAR2B,PRKAR2A,PIP5K1A,NGFR,IKBKB,PSMA5,PSMA3,PSMA2,EGFR,RICTOR,STAT3,PSMC1,AP2M1,SQSTM1,NRG1,MET,SRC,CRKL,TP53,PTK2,GRB2,CALM1,AP2A1,AKAP13,GAB1,ARHGEF2,ARHGEF7,KITLG,PEBP1,ADAM17,RAF1,ARHGEF26,RTN4,ECT2,GSK3B,GSK3A,YWHAB,ARHGEF12,ARHGEF17,ARHGEF16,RALA,PSMB10,MAPK14,TRIO,PRKCD,ADCY9,UBA52,MAPKAP1,ABR,BRAP,BRAF,RET,CLTC,CLTA,PEA15,FGD1,ADCY6,FGD3,IRS1,IRS2,MAP2K2,OBSCN,ITPR3,NF1,SOS1,HDAC2,HDAC1,PTPN11,MTOR,RPS6KA3,SPTBN5,SPTBN1,SPTBN2,VAV2,CAMK4,ARHGDIA,TSC2,MARK3,DLG4,FGFR4,FGFR3,RASA3,AKT1S1,CAMK2D,GNA13,ERBB3,ERBB2,NCAM1,CAMK2G,MAPK3,BAD
Proteoglycans in cancer(K)	0,0189	205	61	1,37E-12	2,15E-10	ARAF,AKT2,FRS2,HSPG2,IQGAP1,FN1,RRAS2,EGFR,FLNA,FLNB,FLNC,STAT3,SLC9A1,ITGB1,ITGB5,ITGB3,ITGAV,PPP1R12A,ITGA2,ITGA5,MET,SRC,EIF4B,PDCD4,ANK3,ANK1,TP53,SDC4,SDC2,PTK2,SDC1,GRB2,GAB1,CTTN,CD44,ACTG1,RAF1,CAV2,CAV1,MSN,ARHGEF12,MAPK14,PRKCB,BRAF,PPP1CB,MAP2K2,RPS6,ITPR3,SOS1,PTPN11,MTOR,EZR,VAV2,CTNNB1,PXN,CAMK2D,ERBB3,ERBB2,CAMK2G,MAPK3,RPS6KB1
Signaling events mediated by Hepatocyte Growth Factor Receptor (c-Met)(N)	0,0074	80	35	4,66E-12	6,29E-10	AKT2,RAP1B,EIF4EBP1,MET,SRC,PTPN1,PTPN2,CRKL,RIN2,PTK2,RANBP10,GRB2,WASL,GAB1,PAK2,EPS15,PAK4,RAF1,DEPTOR,CDH1,MAP2K4,MAP2K2,NUMB,SOS1,PTPN11,MTOR,APC,SH3KBP1,RPTOR,CTNNA1,CTNNB1,PXN,AKT1S1,MAPK3,BAD
Signaling by EGFR(R)	0,0292	317	78	1,16E-11	1,37E-09	ARAF,PSMD8,PSMD2,PSMD1,FRS2,PRKAR1B,PRKAR1A,PSME4,PRKAR2B,PRKAR2A,PIP5K1A,EPS15L1,PSMA5,PSMA3,PSMA2,EGFR,RICTOR,PSMC1,NRG1,MET,SRC,PTPN3,TP53,PTK2,GRB2,CALM1,PTPRK,GAB1,ARHGEF7,EPS15,KITLG,PEBP1,ADAM10,ADAM17,RAF1,GSK3B,GSK3A,YWHAB,PSMB10,PRKCD,ADCY9,UBA52,MAPKAP1,BRAP,BRAF,RET,PEA15,ADCY6,IRS1,IRS2,MAP2K2,ITPR3,NF1,SOS1,PTPN11,PTPN12,MTOR,SPTBN5,SPTBN1,SPTBN2,SH3KBP1,CAMK4,TSC2,PXN,MARK3,DLG4,FGFR4,FGFR3,RASA3,AKT1S1,CAMK2D,ERBB3,ERBB2,LRIG1,NCAM1,CAMK2G,MAPK3,BAD
L1CAM interactions(R)	0,0075	82	34	3,81E-11	4,00E-09	DPYSL2,CSNK2A2,EGFR,AP2M1,ITGB1,ITGB3,ITGAV,ITGA2,ITGA5,ITGA9,SRC,ANK1,CSNK2B,NRP1,AP2A1,KIF4A,LAMB1,CLTC,CLTA,MAP2K2,NUMB,RPS6KA3,EZR,SPTBN5,SPTBN1,SPTBN2,VAV2,SDCBP,HSPA8,DLG1,DLG3,DLG4,NCAM1,MAPK3
Signaling by VEGF(R)	0,0264	287	71	7,96E-11	7,49E-09	ARAF,PSMD8,PSMD2,PSMD1,FRS2,PSME4,JUP,SHB,PSMA5,PSMA3,PSMA2,EGFR,RICTOR,PSMC1,ITGB3,ITGAV,HSP90AA1,NRG1,MET,SRC,BAIAP2,PTK2,GRB2,CALM1,NRP1,PAK2,KITLG,PEBP1,RAF1,CAV1,YWHAB,PSMB10,MAPK14,CYFIP1,PRKCB,PRKCD,UBA52,MAPKAP1,BRAP,BRAF,RET,PEA15,ABI1,IRS1,CTNND1,IRS2,MAP2K2,ITPR3,NF1,BRK1,SOS1,MTOR,SPTBN5,SPTBN1,SPTBN2,VAV2,CTNNA1,AXL,PXN,MARK3,DLG4,FGFR4,FGFR3,HSPB1,RASA3,CAMK2D,ERBB3,ERBB2,NCAM1,CAMK2G,MAPK3
ErbB1 downstream signaling(N)	0,0092	100	37	1,23E-10	1,06E-08	USP6NL,IQGAP1,SFN,EGFR,RICTOR,STAT3,SLC9A1,ARPC5,SRC,SRF,BAIAP2,RIN1,GRB2,CALM1,WASL,GAB1,PEBP1,RAF1,YWHAB,YWHAG,RALA,EPS8,PRKCD,MAPKAP1,BRAF,ABI1,MAP2K4,MAP2K2,RPS6,BRK1,SOS1,MTOR,RPS6KA3,VAV2,DIAPH3,MAPK3,BAD
PDGFR-beta signaling pathway(N)	0,011	120	40	4,42E-10	3,45E-08	USP6NL,IQGAP1,SFN,LYN,EIF2AK2,STAT3,RAP1B,SLC9A3R1,ARPC5,ITGB3,ITGAV,SRC,SRF,PTPN1,BAIAP2,PTPN2,ACTN4,YES1,GRB2,WASL,GAB1,CTTN,RAF1,YWHAB,YWHAG,EPS8,PRKCD,BRAF,ABI1,MAP2K4,MAP2K2,MAP2K7,BRK1,SOS1,PTPN11,RPS6KA3,VAV2,ARHGAP35,ARHGDIA,MAPK3
Cellular response to heat stress(R)	0,0072	78	31	7,40E-10	5,33E-08	NUP107,HSPH1,SIRT1,HSP90AB1,HSP90AA1,HSF1,RAE1,RPA2,VCP,NUP155,NUP153,GSK3B,NUP214,NUP210,NUP98,NUP88,ST13,NDC1,HSPA1B,BAG4,BAG3,DNAJC2,DNAJB6,MTOR,RPTOR,RANBP2,HSPA8,AKT1S1,CAMK2D,CAMK2G,MAPK3
Focal adhesion(K)	0,0185	201	54	9,67E-10	6,48E-08	AKT2,FN1,COL1A1,EGFR,FLNA,FLNB,FLNC,RAP1B,ITGB1,ITGB5,ITGB4,ITGB3,ITGAV,PPP1R12A,ITGA4,ITGA3,ITGA2,ITGA6,ITGA5,MET,ITGA9,SRC,CRKL,ACTN4,PTK2,GRB2,PAK6,PAK2,PAK4,ACTG1,RAF1,VCL,CAV2,CAV1,GSK3B,ILK,LAMC2,LAMB1,PRKCB,BRAF,ZYX,TNC,PPP1CB,SOS1,TLN1,PARVA,VAV2,CTNNB1,ARHGAP35,PXN,COL6A3,ERBB2,MAPK3,BAD
rRNA processing(R)	0,0165	179	50	1,16E-09	7,33E-08	LAS1L,RPLP0,TEX10,BYSL,PELP1,NOP56,NOP58,RPL23A,CSNK1D,CSNK1E,PES1,EXOSC2,DKC1,DDX47,UTP18,BMS1,RPL4,RPL30,RPL3,RPL34,RPL21,RPL26,RPL29,RPL28,UBA52,WDR43,RIOK1,RPL12,RPS3,RPL14,RRP36,RPL13,RPS2,RPL15,RPL17,RPL19,RPL10A,RPS8,RPS6,RPSA,RPL13A,KRR1,RPL18A,XRN2,RPS27,RPS20,FBL,NOP2,RPS3A,SENP3
Signaling events mediated by VEGFR1 and VEGFR2(N)	0,0058	63	27	1,92E-09	1,13E-07	IQGAP1,SHB,HSP90AB1,ITGB3,ITGAV,HSP90AA1,SRC,PTPN2,PTK2,GRB2,CALM1,GAB1,PAK2,RAF1,VCL,CAV1,MAPK14,PRKCB,PRKCD,BRAF,MAP2K2,PTPN11,AKAP1,CTNNA1,CTNNB1,PXN,MAPK3
Clathrin-mediated endocytosis(R)	0,0108	117	38	2,39E-09	1,30E-07	TFRC,ITSN1,FNBP1,GAPVD1,EPS15L1,VAMP8,EGFR,AP2M1,ARPC5,AAK1,ARRB1,FCHO2,TGOLN2,APOB,GRB2,AP2A1,WASL,GAK,CTTN,EPS15,IGF2R,FNBP1L,BIN1,UBA52,CLTC,CLTA,LDLRAP1,GPS1,M6PR,SYNJ2,COPS3,DAB2,PIK3C2A,AGFG1,SH3KBP1,TRIP10,HSPA8,REPS1
Eukaryotic Translation Initiation(R)	0,0103	112	37	2,49E-09	1,30E-07	RPLP0,EIF4EBP1,EIF5B,EIF4B,RPL23A,EIF3H,EIF3E,EIF3C,RPL4,RPL30,RPL3,RPL34,RPL21,RPL26,RPL29,RPL28,UBA52,RPL12,RPS3,RPL14,RPL13,RPS2,RPL15,RPL17,RPL19,RPL10A,RPS8,RPS6,RPSA,EIF2B5,EIF4G1,RPL13A,RPL18A,RPS27,RPS20,EIF2S2,RPS3A
Spliceosome(K)	0,0123	134	41	3,03E-09	1,48E-07	SRSF10,SRSF2,SRSF3,SRSF5,SNRNP200,SRSF6,SRSF7,SRSF8,CTNNBL1,SRSF1,U2AF2,HNRNPA1,HNRNPA3,EIF4A3,DHX8,PCBP1,SNW1,PRPF40A,SLU7,RBM25,DDX42,RBM17,NCBP1,TRA2B,TRA2A,HNRNPU,HNRNPM,HNRNPK,HNRNPC,SF3B2,SF3B1,HSPA1B,THOC2,SNRNP27,SNRPC,CHERP,PPIL1,PRPF4,PRPF3,HSPA8,USP39
Nonsense-Mediated Decay (NMD)(R)	0,0099	108	36	3,25E-09	1,53E-07	RPLP0,CASC3,RPL23A,EIF4A3,NCBP1,RPL4,RPL30,RPL3,RPL34,RPL21,RPL26,RPL29,RPL28,UBA52,SMG1,SMG7,RPL12,RPS3,RPL14,RPL13,RPS2,RPL15,RPL17,RPL19,RPL10A,RPS8,RPS6,RPSA,EIF4G1,RPL13A,RPL18A,RPS27,RNPS1,RPS20,DCP1A,RPS3A
Insulin signaling pathway(K)	0,0129	140	42	3,41E-09	1,53E-07	ARAF,AKT2,PHKA1,PHKA2,PRKAB1,PRKAR1B,PRKAR1A,PRKAR2B,PRKAR2A,FASN,IKBKB,EIF4EBP1,PRKAA1,PTPN1,CRKL,PPP1R3D,GRB2,CALM1,RAF1,ACACB,ACACA,GSK3B,BRAF,PYGB,IRS1,IRS4,IRS2,PPP1CB,MAP2K2,RPS6,PHKB,SOS1,MTOR,RPTOR,GYS1,SORBS1,TRIP10,TSC2,FLOT1,MAPK3,BAD,RPS6KB1
CDC42 signaling events(N)	0,0064	70	28	4,22E-09	1,82E-07	IQGAP1,ARPC5,SRC,BAIAP2,YES1,WASL,ARHGEF7,PAK2,PAK4,RAF1,GSK3B,MAPK14,EPS8,BRAF,CDH1,CFL1,MAP2K4,MAP2K7,MTOR,APC,VAV2,CTNNA1,CTNNB1,ARHGDIA,DLG1,DIAPH3,MAPK3,RPS6KB1
Integrin signalling pathway(P)	0,0145	158	45	4,44E-09	1,82E-07	ARAF,FN1,COL1A1,MICALL1,FLNA,FLNB,COL3A1,LIMS1,COL17A1,RAP1B,ARPC5,ITGB1,ITGB5,ITGB4,ITGB3,ITGAV,ITGA4,ITGA3,ITGA2,ITGA6,ITGA5,ITGA9,SRC,CRKL,ACTN4,PTK2,GRB2,ACTG1,RAF1,VCL,CAV1,ARPC5L,BRAF,COL16A1,MAP2K4,MAP2K2,PIK3C2A,SOS1,PTPN12,TLN1,PARVA,PXN,COL6A3,MAPK6,MAPK3
Tight junction(K)	0,0126	137	41	5,58E-09	2,18E-07	F11R,CLDN2,CLDN1,AKT2,CSNK2A2,CLDN4,CLDN3,CLDN8,CLDN7,EPB41L1,EPB41L2,RRAS2,SRC,ZAK,ACTN4,CSNK2B,EPB41,SYMPK,YES1,CTTN,GNAI3,ACTG1,CASK,AMOTL1,PRKCB,PRKCD,PARD3,MAGI3,MPDZ,LLGL1,LLGL2,OCLN,CTNNA1,TJP1,CTNNB1,TJP3,TJP2,MYH14,MYH10,YBX3,MYH9
Signaling by Type 1 Insulin-like Growth Factor 1 Receptor (IGF1R)(R)	0,0235	255	61	5,90E-09	2,18E-07	ARAF,PSMD8,AKT2,PSMD2,PSMD1,FRS2,PRKAB1,PSME4,PSMA5,PSMA3,PSMA2,EGFR,PSMC1,EIF4EBP1,NRG1,MET,PRKAA1,EIF4B,PTK2,GRB2,CALM1,GAB1,KITLG,PEBP1,RAF1,YWHAB,PSMB10,UBA52,BRAP,BRAF,RET,PEA15,LAMTOR1,IRS1,IRS4,IRS2,MAP2K2,RPS6,NF1,EIF4G1,SOS1,PTPN11,MTOR,SPTBN5,SPTBN1,SPTBN2,RPTOR,TSC2,MARK3,DLG4,FGFR4,FGFR3,RASA3,AKT1S1,CAMK2D,ERBB3,ERBB2,NCAM1,CAMK2G,MAPK3,RPS6KB1
Signaling by PDGF(R)	0,0302	328	72	8,28E-09	2,98E-07	ARAF,PSMD8,PSMD2,PSMD1,FRS2,PRKAR1B,PRKAR1A,PSME4,PRKAR2B,PRKAR2A,PIP5K1A,PSMA5,PSMA3,PSMA2,EGFR,RICTOR,STAT3,PSMC1,NRG1,MET,SRC,CRKL,TP53,GRB7,PTK2,GRB2,CALM1,GAB1,KITLG,PEBP1,RAF1,GSK3B,GSK3A,YWHAB,PSMB10,PRKCD,ADCY9,UBA52,MAPKAP1,BRAP,BRAF,RET,PEA15,ADCY6,IRS1,IRS2,MAP2K2,ITPR3,NF1,SOS1,PTPN11,PTPN12,MTOR,SPTBN5,SPTBN1,SPTBN2,CAMK4,TSC2,MARK3,DLG4,FGFR4,FGFR3,RASA3,AKT1S1,COL6A3,CAMK2D,ERBB3,ERBB2,NCAM1,CAMK2G,MAPK3,BAD
Signaling by MET(R)	0,0048	52	23	1,68E-08	5,88E-07	STAT3,RAP1B,ITGB1,ITGA3,ITGA2,MET,SRC,PTPN1,PTPN2,CRKL,PTK2,RANBP10,GRB2,GAB1,EPS15,UBA52,USP8,SOS1,PTPN11,TNS4,TNS3,SH3KBP1,LRIG1
Eukaryotic Translation Elongation(R)	0,0078	85	30	1,91E-08	6,29E-07	RPLP0,EEF2,RPL23A,EEF1D,RPL4,RPL30,RPL3,RPL34,RPL21,RPL26,RPL29,RPL28,UBA52,RPL12,RPS3,RPL14,RPL13,RPS2,RPL15,RPL17,RPL19,RPL10A,RPS8,RPS6,RPSA,RPL13A,RPL18A,RPS27,RPS20,RPS3A
DAP12 interactions(R)	0,0297	323	69	4,72E-08	1,51E-06	ARAF,PSMD8,PSMD2,PSMD1,FRS2,PRKAR1B,PRKAR1A,PSME4,PRKAR2B,PRKAR2A,PIP5K1A,PSMA5,PSMA3,PSMA2,EGFR,RICTOR,PSMC1,NRG1,MET,TP53,PTK2,GRB2,CALM1,GAB1,KITLG,PEBP1,RAF1,GSK3B,GSK3A,YWHAB,PSMB10,PRKCD,ADCY9,UBA52,MAPKAP1,BRAP,BRAF,RET,PEA15,ADCY6,IRS1,IRS2,MAP2K2,ITPR3,NF1,SOS1,PTPN11,MTOR,HLA-C,HLA-E,SPTBN5,SPTBN1,SPTBN2,VAV2,CAMK4,TSC2,MARK3,DLG4,FGFR4,FGFR3,RASA3,AKT1S1,CAMK2D,ERBB3,ERBB2,NCAM1,CAMK2G,MAPK3,BAD
Integrins in angiogenesis(N)	0,0043	47	21	5,87E-08	1,76E-06	F11R,FN1,ITGB3,ITGAV,HSP90AA1,SRC,PTK2,SDC1,MFGE8,VCL,ILK,CSF1,IRS1,PIK3C2A,PTPN11,TLN1,PXN,PI4KA,TGFBR2,MAPK3,RPS6KB1
Apoptotic execution phase(R)	0,0043	47	21	5,87E-08	1,76E-06	FNTA,STK26,DNM1L,DSP,DSG2,PTK2,PAK2,H1F0,LMNB1,PLEC,ADD1,VIM,PRKCD,CDH1,APC,OCLN,LMNA,TJP1,CTNNB1,TJP2,CASP6
Eukaryotic Translation Termination(R)	0,0079	86	29	8,41E-08	2,44E-06	RPLP0,APEH,RPL23A,RPL4,RPL30,RPL3,RPL34,RPL21,RPL26,RPL29,RPL28,UBA52,RPL12,RPS3,RPL14,RPL13,RPS2,RPL15,RPL17,RPL19,RPL10A,RPS8,RPS6,RPSA,RPL13A,RPL18A,RPS27,RPS20,RPS3A
Signaling by SCF-KIT(R)	0,0267	290	63	1,03E-07	2,87E-06	ARAF,PSMD8,PSMD2,PSMD1,FRS2,PSME4,PIP5K1A,LYN,PSMA5,PSMA3,PSMA2,EGFR,RICTOR,STAT3,PSMC1,NRG1,MET,SRC,TP53,GRB7,YES1,PTK2,GRB2,CALM1,GAB1,KITLG,PEBP1,RAF1,GSK3B,GSK3A,YWHAB,PSMB10,UBA52,MAPKAP1,BRAP,BRAF,RET,PEA15,IRS1,IRS2,MAP2K2,NF1,SOS1,PTPN11,MTOR,CHEK1,SPTBN5,SPTBN1,SPTBN2,TSC2,MARK3,DLG4,FGFR4,FGFR3,RASA3,AKT1S1,CAMK2D,ERBB3,ERBB2,NCAM1,CAMK2G,MAPK3,BAD
Adherens junction(K)	0,0066	72	26	1,07E-07	2,89E-06	CSNK2A2,IQGAP1,EGFR,MAP3K7,MET,SRC,PTPN1,BAIAP2,ACTN4,CSNK2B,YES1,PTPRM,WASL,ACTG1,VCL,PARD3,CTNND1,CDH1,CTNNA1,SORBS1,TJP1,CTNNB1,LMO7,TGFBR2,ERBB2,MAPK3
ErbB signaling pathway(K)	0,0081	88	29	1,35E-07	3,63E-06	ARAF,AKT2,EGFR,EIF4EBP1,NRG1,SRC,CRKL,PTK2,GRB2,GAB1,PAK6,PAK2,PAK4,RAF1,GSK3B,PRKCB,BRAF,MAP2K4,MAP2K2,MAP2K7,SOS1,MTOR,CAMK2D,ERBB3,ERBB2,CAMK2G,MAPK3,BAD,RPS6KB1
mTOR signaling pathway(N)	0,0059	64	24	1,68E-07	4,23E-06	SFN,IKBKB,RICTOR,EIF4EBP1,EEF2,EIF4B,PDCD4,RAF1,YWHAB,YWHAG,BNIP3,MAPKAP1,BRAF,IRS1,DEPTOR,MAP2K2,CLIP1,MTOR,PML,RPTOR,TSC2,AKT1S1,MAPK3,RPS6KB1
EPH-Ephrin signaling(R)	0,0082	89	29	1,69E-07	4,23E-06	ITSN1,MYL6,LYN,SHB,AP2M1,ARPC5,HSP90AA1,SRC,EPHB4,EPHB3,EPHA4,EPHA2,SDC2,YES1,PTK2,AP2A1,WASL,GIT1,ARHGEF7,ACTG1,ADAM10,CFL1,VAV2,EFNB2,SDCBP,EFNB1,MYH14,MYH10,MYH9
Signaling by Insulin receptor(R)	0,0255	277	60	2,27E-07	5,46E-06	ARAF,PSMD8,AKT2,PSMD2,PSMD1,FRS2,PRKAB1,PSME4,PSMA5,PSMA3,PSMA2,EGFR,PSMC1,EIF4EBP1,NRG1,MET,PRKAA1,EIF4B,PTK2,GRB2,CALM1,GAB1,KITLG,PEBP1,RAF1,YWHAB,PSMB10,UBA52,BRAP,BRAF,RET,PEA15,LAMTOR1,IRS1,IRS2,MAP2K2,RPS6,NF1,EIF4G1,SOS1,PTPN11,MTOR,SPTBN5,SPTBN1,SPTBN2,RPTOR,TSC2,MARK3,DLG4,FGFR4,FGFR3,RASA3,AKT1S1,CAMK2D,ERBB3,ERBB2,NCAM1,CAMK2G,MAPK3,RPS6KB1
Translocation of GLUT4 to the plasma membrane(R)	0,0048	52	21	3,01E-07	7,23E-06	AKT2,PRKAB1,SFN,LNPEP,STX4,SNAP23,MYO1C,MYO5A,CALM1,KIF3A,YWHAB,YWHAG,TBC1D4,RALA,EXOC1,RALGAPA2,RAB10,KIFAP3,STXBP3,C2CD5,RAB8A
RET signaling(R)	0,0204	222	51	3,43E-07	7,88E-06	ARAF,PSMD8,PSMD2,PSMD1,FRS2,PSME4,PSMA5,PSMA3,PSMA2,EGFR,PSMC1,RAP1GAP,NRG1,MET,SRC,GRB7,PTK2,GRB2,CALM1,GAB1,KITLG,PEBP1,RAF1,YWHAB,SHANK3,PSMB10,UBA52,BRAP,BRAF,RET,PEA15,IRS1,IRS2,MAP2K2,NF1,SOS1,PTPN11,SPTBN5,SPTBN1,SPTBN2,MARK3,DLG4,FGFR4,FGFR3,RASA3,CAMK2D,ERBB3,ERBB2,NCAM1,CAMK2G,MAPK3
SRP-dependent cotranslational protein targeting to membrane(R)	0,0095	103	31	3,48E-07	8,01E-06	RPLP0,SRP9,RPL23A,SRP14,RPL4,RPL30,RPL3,RPL34,RPL21,RPL26,RPL29,RPL28,UBA52,RPL12,RPS3,RPL14,RPL13,RPS2,RPL15,RPL17,RPL19,RPL10A,RPS8,RPS6,RPSA,RPL13A,RPL18A,RPS27,RPS20,SEC61B,RPS3A
Arrhythmogenic right ventricular cardiomyopathy (ARVC)(K)	0,0066	72	25	3,79E-07	8,35E-06	JUP,PKP2,ITGB1,ITGB5,ITGB4,ITGB3,ITGAV,DSP,ITGA4,ITGA3,ITGA2,DSG2,ITGA6,ITGA5,ITGA9,ACTN4,ACTG1,ATP2A2,GJA1,SLC8A1,CACNG4,DAG1,LMNA,CTNNA1,CTNNB1
Signaling events mediated by focal adhesion kinase(N)	0,0055	60	22	7,76E-07	1,71E-05	RAP1B,ITGB1,ITGB5,ITGAV,ITGA5,SRC,GRB7,YES1,PTK2,GRB2,WASL,ARHGEF7,SH3GL1,RAF1,VCL,ASAP1,BRAF,MAP2K4,SOS1,TLN1,ARHGAP35,PXN
EGFR tyrosine kinase inhibitor resistance(K)	0,0075	81	26	9,47E-07	1,99E-05	ARAF,AKT2,EGFR,STAT3,EIF4EBP1,NRG1,MET,SRC,GRB2,GAB1,RAF1,GSK3B,PRKCB,BRAF,MAP2K2,RPS6,NF1,SOS1,MTOR,AXL,FGFR3,ERBB3,ERBB2,MAPK3,BAD,RPS6KB1
Selenoamino acid metabolism(R)	0,0095	103	30	1,03E-06	2,17E-05	RPLP0,RPL23A,RPL4,RPL30,RPL3,RPL34,EPRS,RPL21,RPL26,RPL29,RPL28,UBA52,RPL12,RPS3,RPL14,RPL13,RPS2,RPL15,RPL17,RPL19,RPL10A,RPS8,RPS6,RPSA,IARS,RPL13A,RPL18A,RPS27,RPS20,RPS3A
Mitotic G2-G2/M phases(R)	0,016	174	42	1,09E-06	2,18E-05	PSMD8,PSMD2,PSMD1,GTSE1,PSME4,OPTN,PRKAR2B,PSMA5,PSMA3,PSMA2,CENPF,CENPJ,PSMC1,NEK2,FGFR1OP,TUBGCP3,HSP90AB1,NUMA1,HSP90AA1,PPP1R12A,CSNK1D,CSNK1E,TP53,CETN2,PCM1,DYNC1H1,NEDD1,YWHAG,PSMB10,AURKA,UBA52,PPP1CB,PHLDA1,CEP131,CDK7,CDK1,SKP1,TPX2,RAB8A,TUBB,CKAP5,CLASP1
Nectin adhesion pathway(N)	0,0028	30	15	1,14E-06	2,29E-05	F11R,CLDN1,IQGAP1,RAP1B,ITGB3,ITGAV,SRC,PTK2,PTPRM,CDH1,TLN1,VAV2,CTNNA1,CTNNB1,PVR
Bacterial invasion of epithelial cells(K)	0,0072	78	25	1,56E-06	2,96E-05	CD2AP,FN1,ARPC5,ITGB1,ITGA5,MET,SRC,CRKL,PTK2,WASL,GAB1,CTTN,ACTG1,VCL,ARHGEF26,CAV2,CAV1,ILK,ARPC5L,CLTC,CLTA,CDH1,CTNNA1,CTNNB1,PXN
Signaling by Leptin(R)	0,0192	209	47	1,73E-06	3,28E-05	ARAF,PSMD8,PSMD2,PSMD1,FRS2,PSME4,PSMA5,PSMA3,PSMA2,EGFR,STAT3,PSMC1,NRG1,MET,PTK2,GRB2,CALM1,KITLG,PEBP1,RAF1,YWHAB,PSMB10,UBA52,BRAP,BRAF,RET,PEA15,IRS1,IRS2,MAP2K2,NF1,SOS1,PTPN11,SPTBN5,SPTBN1,SPTBN2,MARK3,DLG4,FGFR4,FGFR3,RASA3,CAMK2D,ERBB3,ERBB2,NCAM1,CAMK2G,MAPK3
Aurora B signaling(N)	0,0037	40	17	2,01E-06	3,62E-05	NCAPG,CDCA8,NSUN2,PSMA3,INCENP,CENPA,KIF20A,RACGAP1,KIF23,NPM1,PEBP1,AURKA,VIM,STMN1,H3F3A,NCL,CBX5
Integrin-linked kinase signaling(N)	0,0041	45	18	2,35E-06	4,23E-05	IQGAP1,RICTOR,LIMS1,NACA,HSP90AA1,PPP1R12A,CDC37,ARHGEF7,GSK3B,ILK,AURKA,ZYX,TNS1,PARVA,PARP1,CTNNB1,PXN,CKAP5
RAF/MAP kinase cascade(R)	0,0185	201	45	3,19E-06	5,46E-05	ARAF,PSMD8,PSMD2,PSMD1,FRS2,PSME4,PSMA5,PSMA3,PSMA2,EGFR,PSMC1,NRG1,MET,PTK2,GRB2,CALM1,KITLG,PEBP1,RAF1,YWHAB,PSMB10,UBA52,BRAP,BRAF,RET,PEA15,IRS1,IRS2,MAP2K2,NF1,SOS1,SPTBN5,SPTBN1,SPTBN2,MARK3,DLG4,FGFR4,FGFR3,RASA3,CAMK2D,ERBB3,ERBB2,NCAM1,CAMK2G,MAPK3
ErbB2/ErbB3 signaling events(N)	0,0034	37	16	3,21E-06	5,46E-05	STAT3,NRG1,SRC,GRB2,RAF1,USP8,MAP2K2,NF2,SOS1,PTPN11,MTOR,DOCK7,ERBB3,ERBB2,MAPK3,BAD
Factors involved in megakaryocyte development and platelet production(R)	0,0101	110	30	3,73E-06	6,34E-05	PRKAR1B,PRKAR1A,GATA3,PRKAR2B,PRKAR2A,HIST2H3A,TP53,RACGAP1,RCOR1,KIF11,KIF15,AKAP10,KIF23,KIF3A,KIF4A,H3F3A,EHD1,EHD2,KLC1,KLC2,CDK5,SIN3A,HDAC2,HDAC1,KIFAP3,AKAP1,CAPZA1,CAPZA2,CBX5,KIF18A
ERCC6 (CSB) and EHMT2 (G9a) positively regulate rRNA expression(R)	0,0017	18	11	4,76E-06	8,10E-05	MBD3,HIST2H3A,CHD4,CHD3,MTA2,MTA3,H3F3A,HDAC2,HDAC1,HIST1H4A,CBX3
SUMOylation(R)	0,0098	106	29	5,07E-06	8,11E-05	NUP107,CDCA8,INCENP,SUMO1,TOP1,NOP58,TP53,CETN2,RAE1,TFAP2C,NUP155,NUP153,AURKA,NUP214,NUP210,NUP98,NUP88,PHC3,HNRNPK,HNRNPC,NDC1,PML,TP53BP1,TOP2A,TOP2B,PARP1,SP100,RANBP2,CBX4
a6b1 and a6b4 Integrin signaling(N)	0,0032	35	15	7,15E-06	1,08E-04	SFN,EGFR,COL17A1,ITGB1,ITGB4,ITGA6,MET,MST1R,GRB2,YWHAB,YWHAG,CDH1,ERBB3,ERBB2,RPS6KB1
Class I PI3K signaling events mediated by Akt(N)	0,0032	35	15	7,15E-06	1,08E-04	AKT2,SFN,RICTOR,HSP90AA1,SRC,RAF1,GSK3B,GSK3A,YWHAB,YWHAG,TBC1D4,PRKDC,MAPKAP1,MTOR,BAD
Internalization of ErbB1(N)	0,0032	35	15	7,15E-06	1,08E-04	ITSN1,ZFYVE28,EGFR,SRC,PTK2,GRB2,ARHGEF7,EPS15,RAF1,TSG101,USP8,SOS1,SH3KBP1,CHMP3,LRIG1
RNA Polymerase II Transcription(R)	0,0133	144	35	7,23E-06	1,08E-04	DDX39A,ZC3H11A,NELFE,LEO1,SRSF11,CASC3,SRSF2,SRSF3,SRSF5,SRSF6,SRSF7,SRSF1,MLLT1,U2AF2,CCNK,EIF4A3,CTR9,SYMPK,PAF1,SLU7,SUPT5H,AFF4,NCBP1,TAF12,SSRP1,SUPT16H,THOC2,THOC5,THOC7,CDK7,SRRM1,CSTF3,RNPS1,CPSF7,IWS1
Cell junction organization(R)	0,0064	70	22	8,69E-06	1,30E-04	F11R,JUP,FERMT2,FLNA,FLNC,LIMS1,COL17A1,ITGB1,ITGB4,ITGA6,ILK,LAMC2,PLEC,PARD3,CTNND1,CDH6,CDH1,PARVA,CTNNA1,PVR,PXN,CDH10
Semaphorin interactions(R)	0,006	65	21	9,21E-06	1,38E-04	DPYSL2,DPYSL3,MYL6,ITGB1,HSP90AB1,HSP90AA1,MET,NRP1,PAK2,GSK3B,ARHGEF12,CFL1,PLXNA2,PLXNA1,CDK5,TLN1,ARHGAP35,MYH14,MYH10,MYH9,ERBB2
Thyroid hormone signaling pathway(K)	0,0109	118	30	1,40E-05	2,09E-04	AKT2,MED12,MED14,MED13,SLC9A1,ITGB3,ITGAV,SRC,TP53,NCOA3,ACTG1,RAF1,GSK3B,ATP2A2,TBC1D4,PRKCB,PLCD1,MAP2K2,NOTCH2,SIN3A,HDAC2,HDAC1,MTOR,PFKP,CTNNB1,TSC2,SLC2A1,ATP1A1,MAPK3,BAD
Stabilization and expansion of the E-cadherin adherens junction(N)	0,0039	42	16	1,51E-05	2,11E-04	MYO6,EGFR,STX4,MET,EPHA2,GIT1,PLEKHA7,VCL,ABI1,ZYX,CTNND1,CDH1,LIMA1,CTNNA1,CTNNB1,CAMSAP3
Endometrial cancer(K)	0,0048	52	18	1,61E-05	2,25E-04	ARAF,AKT2,EGFR,TP53,GRB2,RAF1,GSK3B,ILK,BRAF,CDH1,MAP2K2,SOS1,APC,CTNNA1,CTNNB1,ERBB2,MAPK3,BAD
EGF receptor (ErbB1) signaling pathway(N)	0,0027	29	13	1,81E-05	2,53E-04	EGFR,STAT3,SRC,PTPN1,PTK2,GRB2,WASL,GAB1,GNAI3,SOS1,PTPN11,TLN1,MAPK3
Regulation of RAC1 activity(N)	0,0035	38	15	1,84E-05	2,57E-04	PREX1,BCR,ARHGAP1,RACGAP1,ARHGEF2,ARHGEF7,TRIO,EPS8,ABR,ABI1,SOS1,VAV2,ARHGDIA,RALBP1,ARHGAP17
Cell Cycle Checkpoints(R)	0,0151	164	37	1,98E-05	2,57E-04	PSMD8,PSMD2,PSMD1,GTSE1,PSME4,SFN,ATRIP,PSMA5,PSMA3,PSMA2,PSMC1,ANAPC2,SUMO1,CDC20,CDC23,CDC26,TP53,BABAM1,UIMC1,RPA2,YWHAB,YWHAG,PSMB10,UBA52,CDC6,NBN,ORC6,ORC1,CDK1,MCM3,MCM6,MCM2,CHEK1,TP53BP1,HIST1H4A,H2AFX,HIST3H3
NCAM signaling for neurite out-growth(R)	0,0213	231	47	2,17E-05	2,81E-04	ARAF,PSMD8,PSMD2,PSMD1,FRS2,PSME4,PSMA5,PSMA3,PSMA2,EGFR,PSMC1,NRG1,MET,SRC,PTK2,GRB2,CALM1,KITLG,PEBP1,RAF1,YWHAB,PSMB10,UBA52,BRAP,BRAF,RET,PEA15,IRS1,IRS2,MAP2K2,NF1,SOS1,SPTBN5,SPTBN1,SPTBN2,MARK3,DLG4,FGFR4,FGFR3,RASA3,COL6A3,CAMK2D,ERBB3,ERBB2,NCAM1,CAMK2G,MAPK3
Validated targets of C-MYC transcriptional activation(N)	0,0069	75	22	2,44E-05	3,14E-04	TFRC,ACTL6A,HSP90AA1,GAPDH,RCC1,TP53,PTMA,NPM1,HUWE1,HMGA1,CAD,TAF12,ENO1,MTDH,TK1,NBN,NCL,EIF4G1,UBTF,HSPA4,SLC2A1,HSPD1
IGF1 pathway(N)	0,0028	30	13	2,55E-05	3,14E-04	PTPN1,CRKL,PTK2,GRB2,RAF1,PRKCD,IRS1,IRS2,SOS1,PTPN11,PXN,BAD,RPS6KB1
Leukocyte transendothelial migration(K)	0,0107	116	29	2,60E-05	3,14E-04	F11R,CLDN2,CLDN1,CLDN4,CLDN3,CLDN8,CLDN7,RAP1B,ITGB1,ITGA4,ICAM1,ACTN4,PTK2,GNAI3,ACTG1,VCL,MSN,MAPK14,PRKCB,CTNND1,PTPN11,EZR,OCLN,VAV2,CXCR4,CTNNA1,CTNNB1,ARHGAP35,PXN
RAC1 signaling pathway(N)	0,005	54	18	2,62E-05	3,14E-04	IQGAP1,PIP5K1A,STAT3,ARPC5,BAIAP2,RACGAP1,PAK2,MAPK14,ABI2,ABI1,CDH1,CFL1,MAP2K4,MAP2K7,BRK1,CTNNA1,CTNNB1,ARHGDIA

Degradation of beta catenin(N)	0,0017	18	10	2,84E-05	3,41E-04	LRP6,CSNK1D,CSNK1E,GSK3B,GSK3A,CSNK1A1,DVL3,APC,SKP1,CTNNB1
ECM-receptor interaction(K)	0,0075	82	23	3,18E-05	3,82E-04	HSPG2,FN1,COL1A1,ITGB1,ITGB5,ITGB4,ITGB3,ITGAV,ITGA4,ITGA3,ITGA2,ITGA6,ITGA5,ITGA9,SDC4,SDC1,CD47,CD44,LAMC2,LAMB1,TNC,DAG1,COL6A3
Epstein-Barr virus infection(K)	0,0188	204	42	4,49E-05	5,39E-04	PSMD8,AKT2,PSMD2,PSMD1,CSNK2A2,SND1,LYN,EIF2AK2,IKBKB,STAT3,PSMC1,MAP3K7,SPN,ICAM1,CSNK2B,TP53,SNW1,PTMA,CD44,GSK3B,YWHAB,TNFAIP3,YWHAG,DDX58,MAPK14,VIM,NUP214,NCOR2,HSPA1B,MAP2K4,MAP2K7,CDK1,TRAF2,POLR1D,HDAC2,HDAC1,POLR3E,HLA-C,HLA-A,HLA-E,HSPA8,HSPB1
Syndecan-2-mediated signaling events(N)	0,0029	32	13	4,89E-05	5,86E-04	FN1,ITGB1,ITGA2,SRC,EPB41,SDC2,CASK,CAV2,PRKCD,NF1,EZR,SDCBP,MAPK3
Neurotrophin signaling pathway(K)	0,0111	121	29	5,41E-05	6,21E-04	AKT2,FRS2,NGFR,IKBKB,RAP1B,CRKL,TP53,GRB2,CALM1,GAB1,SORT1,RAF1,PSEN1,GSK3B,MAPK14,PRKCD,BRAF,IRS1,MAP2K2,MAP2K7,SOS1,PTPN11,RPS6KA3,CAMK4,ARHGDIA,CAMK2D,CAMK2G,MAPK3,BAD
FGF signaling pathway(N)	0,0043	47	16	5,65E-05	6,21E-04	FRS2,SRC,SDC2,GRB2,GAB1,CTTN,PAK4,SSH1,CTNND1,CDH1,SOS1,PTPN11,FGFR4,FGFR3,NCAM1,MAPK3
Endosomal Sorting Complex Required For Transport (ESCRT)(R)	0,0026	28	12	5,72E-05	6,29E-04	VPS37D,TSG101,UBA52,VPS28,VPS4B,VPS4A,CHMP4C,CHMP4B,CHMP2B,CHMP2A,CHMP3,CHMP5
Gap junction trafficking and regulation(R)	0,0008	9	7	5,79E-05	6,37E-04	MYO6,AP2M1,SRC,GJA1,GJB1,DAB2,TJP1
Neutrophil degranulation(R)	0,0362	393	68	6,08E-05	6,68E-04	PSMD2,PSMD1,IQGAP1,JUP,PGAM1,VAMP8,PSMA5,PSMA2,PAFAH1B2,APEH,TOM1,TICAM2,IST1,S100A9,RAP1B,ARPC5,EEF2,HSP90AB1,SNAP23,PGM2,ITGAV,DSP,HSP90AA1,CPNE1,CSNK2B,DSN1,XRCC6,CAP1,CD47,CD44,DYNC1H1,HUWE1,SRP14,OSTF1,CD97,ADAM10,PPIA,VCL,VCP,PSEN1,IGF2R,MAPK14,CYFIP1,PRKCD,DYNC1LI1,S100A11,CDK13,PYGB,GPI,ANXA2,LAMTOR1,HSPA1B,RAB31,RAB5C,UBR4,PKM,DNAJC5,RAB10,IMPDH2,RAB18,FTH1,HLA-C,HLA-A,TUBB,ALDOA,SVIP,SDCBP,HSPA8
EPHB forward signaling(N)	0,0035	38	14	7,14E-05	7,63E-04	ITSN1,RAP1B,SRC,EPHB4,EPHB3,GRB7,PTK2,GRB2,WASL,MAP4K4,PXN,EFNB2,EFNB1,MAPK3
Posttranslational regulation of adherens junction stability and dissassembly(N)	0,0044	48	16	7,19E-05	7,63E-04	IQGAP1,JUP,EGFR,DSP,MET,SRC,PTPN1,RIN2,ADAM10,RET,CTNND1,CDH1,ROBO1,CTNNA1,CTNNB1,GNA13
Signaling by ERBB2(R)	0,004	43	15	7,30E-05	7,63E-04	EGFR,HSP90AA1,NRG1,SRC,STUB1,CDC37,GRB7,GRB2,GAB1,UBA52,USP8,SOS1,PTPN12,ERBB3,ERBB2
LKB1 signaling events(N)	0,004	43	15	7,30E-05	7,63E-04	SFN,STK26,HSP90AA1,CDC37,TP53,GSK3B,YWHAB,YWHAG,MTOR,EZR,RPTOR,TSC2,MARK4,MARK2,AKT1S1
Regulation of mRNA stability by proteins that bind AU-rich elements(R)	0,008	87	23	7,63E-05	7,63E-04	PSMD8,PSMD2,PSMD1,PSME4,PSMA5,PSMA3,PSMA2,PSMC1,EXOSC2,KHSRP,YWHAB,PSMB10,MAPK14,NUP214,PRKCD,UBA52,HNRNPD,HSPA1B,EIF4G1,DCP1A,ZFP36L1,HSPA8,HSPB1
Mitotic Prometaphase(R)	0,0091	99	25	7,77E-05	7,77E-04	NUP107,CSNK2A2,NCAPG,CDCA8,INCENP,CENPA,CENPF,TAOK1,CASC5,CDC20,CSNK2B,DSN1,ERCC6L,PDS5B,NUP98,NUF2,NUDC,CLIP1,CDK1,RPS27,RANBP2,KIF18A,CKAP5,CLASP1,CLASP2
VEGFR3 signaling in lymphatic endothelium(N)	0,0023	25	11	9,16E-05	9,16E-04	FN1,COL1A1,ITGB1,ITGA4,ITGA2,ITGA5,GRB2,MAPK14,MAP2K4,SOS1,MAPK3
Axon guidance(K)	0,0163	177	37	9,25E-05	9,25E-04	DPYSL2,ITGB1,MET,SRC,EPHB4,EPHB3,EPHA4,EPHA2,PTK2,NRP1,GNAI3,PAK6,PAK2,PAK4,RAF1,SSH3,SSH1,GSK3B,ILK,ARHGEF12,PARD3,CFL2,CFL1,PLXNA2,PLXNA1,SRGAP2,SRGAP1,CDK5,BMPR2,PTPN11,ROBO1,CXCR4,EFNB2,EFNB1,CAMK2D,CAMK2G,MAPK3
Signaling events regulated by Ret tyrosine kinase(N)	0,0036	39	14	9,33E-05	9,33E-04	FRS2,SRC,GRB7,PTK2,GRB2,GAB1,SHANK3,RET,IRS1,IRS2,SOS1,PTPN11,PXN,MAPK3
Mitotic Metaphase and Anaphase(R)	0,0151	164	35	9,58E-05	9,58E-04	NUP107,PSMD8,PSMD2,PSMD1,PSME4,CDCA8,PSMA5,PSMA3,PSMA2,INCENP,CENPA,CENPF,TAOK1,PSMC1,CASC5,ANAPC2,CDC20,CDC23,CDC26,DSN1,ERCC6L,PDS5B,PSMB10,NUP98,UBA52,NUF2,NUDC,CLIP1,RPS27,LMNA,RANBP2,KIF18A,CKAP5,CLASP1,CLASP2

Supplementary Table 3. List of a subpopulation of the TNBC- and HER2-EV subtype specific signatures quantified in human serum-derived EVs.

Gene names	Intensity TNBC_1	Intensity TNBC_2	Intensity TNBC_3	Intensity TNBC_4	Intensity TNBC_5	Intensity HER2_1	Intensity HER2_2	Intensity HER2_3	Intensity HER2_4	Intensity HER2_5	Intensity healthy_1	Intensity healthy_2	Intensity healthy_3	Intensity healthy_4	Intensity healthy_5	Intensity CRC_1	Intensity CRC_2	Intensity CRC_3	Intensity CRC_4	Intensity CRC_5	Intensity NSCLC_1	Intensity NSCLC_2	Intensity NSCLC_3	Intensity NSCLC_4	Intensity NSCLC_5
NRP1	25,02606	NaN	26,3014	NaN	NaN	NaN	NaN	NaN	NaN	NaN	NaN	NaN	NaN	NaN	NaN	NaN	23,87624	25,77623	25,55403	NaN	NaN	NaN	NaN	NaN	NaN
ACSL4	25,16196	NaN	NaN	25,73547	NaN	24,42304	25,1495	NaN	NaN	NaN	NaN	25,92846	NaN	25,46476	NaN	NaN	NaN	NaN	NaN	NaN	NaN	NaN	NaN	26,76572	NaN
PLAU	NaN	NaN	NaN	25,00642	NaN	NaN	NaN	NaN	NaN	NaN	NaN	NaN	NaN	NaN	NaN	NaN	NaN	NaN	NaN	NaN	NaN	NaN	24,84245	NaN	NaN
PABPC1	NaN	25,08869	NaN	26,19691	NaN	21,98402	NaN	NaN	24,63029	NaN	NaN	NaN	NaN	NaN	NaN	NaN	NaN	NaN	NaN	NaN	NaN	NaN	NaN	24,48155	NaN
EPHA2	26,30966	NaN	26,41917	NaN	NaN	24,54239	NaN	NaN	26,30156	25,30163	26,93775	NaN	NaN	NaN	24,78786	NaN	NaN	NaN	NaN	26,65608	NaN	NaN	NaN	23,91771	NaN
DNAJA1	26,24131	NaN	26,36264	NaN	26,85567	NaN	25,12609	NaN	NaN	NaN	26,01329	NaN	NaN	25,96585	24,4829	NaN	25,07382	NaN	24,90035	25,87159	NaN	NaN	NaN	NaN	NaN
PABPC4	25,07594	NaN	25,11319	NaN	19,61047	NaN	NaN	NaN	NaN	NaN	NaN	NaN	24,46619	25,42002	NaN	25,16921	24,98251	NaN	24,3686	NaN	NaN	NaN	NaN	NaN	25,85436
ADAM9	NaN	24,63771	NaN	28,32307	NaN	NaN	NaN	NaN	NaN	NaN	NaN	27,58628	NaN	NaN	NaN	26,07198	NaN	NaN	NaN	NaN	27,08071	27,36018	NaN	NaN	26,82043
CSPG4	25,03976	NaN	25,75218	NaN	26,00472	NaN	25,36088	NaN	NaN	NaN	NaN	NaN	NaN	NaN	NaN	NaN	NaN	NaN	NaN	NaN	NaN	NaN	24,44638	27,12849	NaN
CCDC50	NaN	21,40603	NaN	NaN	NaN	NaN	NaN	NaN	NaN	NaN	NaN	NaN	21,36137	NaN	NaN	NaN	NaN	NaN	NaN	22,3165	NaN	NaN	NaN	NaN	NaN
MITD1	NaN	NaN	25,0471	NaN	NaN	NaN	NaN	NaN	NaN	NaN	NaN	NaN	NaN	NaN	NaN	NaN	NaN	NaN	NaN	NaN	NaN	NaN	NaN	NaN	NaN
MOV10	NaN	NaN	NaN	23,1823	NaN	NaN	NaN	NaN	26,86667	NaN	NaN	NaN	NaN	NaN	NaN	NaN	NaN	NaN	NaN	NaN	NaN	NaN	NaN	NaN	NaN
MKRN1	NaN	NaN	28,35502	NaN	NaN	NaN	NaN	NaN	NaN	NaN	NaN	NaN	NaN	NaN	NaN	NaN	NaN	NaN	NaN	NaN	NaN	NaN	NaN	NaN	NaN
HEG1	NaN	NaN	NaN	NaN	23,75898	NaN	NaN	NaN	NaN	NaN	NaN	NaN	NaN	NaN	NaN	NaN	NaN	NaN	NaN	NaN	NaN	NaN	23,87202	NaN	NaN
EIF3F	NaN	NaN	NaN	NaN	NaN	NaN	NaN	NaN	22,78692	NaN	NaN	NaN	NaN	NaN	NaN	NaN	NaN	NaN	NaN	NaN	NaN	NaN	NaN	NaN	NaN
CASK	NaN	NaN	25,63032	NaN	24,52928	NaN	NaN	NaN	25,9896	NaN	NaN	NaN	27,85816	NaN	NaN	NaN	24,90196	NaN	NaN	NaN	NaN	NaN	25,0618	NaN	23,21886
EIF3H	NaN	27,04708	NaN	27,7107	27,09909	NaN	NaN	28,854	28,5823	NaN	NaN	NaN	NaN	NaN	NaN	NaN	NaN	NaN	NaN	NaN	25,96798	NaN	NaN	NaN	NaN
AHSA1	NaN	NaN	NaN	NaN	NaN	NaN	NaN	24,92184	NaN	23,98954	NaN	NaN	NaN	NaN	NaN	NaN	NaN	NaN	NaN	NaN	NaN	NaN	NaN	NaN	NaN
ERBB2	NaN	NaN	NaN	26,18946	NaN	NaN	NaN	26,09273	26,97727	26,82347	24,22685	24,19731	NaN	24,68998	NaN	NaN	NaN	NaN	NaN	NaN	25,23436	NaN	NaN	27,96168	26,40863
PRKCB	NaN	NaN	NaN	NaN	NaN	NaN	NaN	NaN	NaN	24,84532	NaN	23,40717	NaN	NaN	NaN	NaN	NaN	NaN	NaN	NaN	22,86321	NaN	NaN	NaN	NaN
EEF1G	NaN	NaN	NaN	26,61863	NaN	NaN	24,57259	NaN	23,49911	NaN	NaN	NaN	NaN	NaN	23,48124	NaN	23,38284	NaN	NaN	NaN	NaN	NaN	NaN	NaN	NaN
PIK3R1	NaN	NaN	NaN	24,66237	NaN	NaN	NaN	NaN	25,23809	NaN	NaN	NaN	NaN	NaN	NaN	NaN	23,36546	NaN	NaN	NaN	24,30987	NaN	NaN	NaN	NaN
STIP1	NaN	NaN	23,63898	24,49387	25,45795	24,31741	NaN	25,46756	NaN	NaN	26,85317	NaN	24,503	25,15592	24,83316	24,8541	NaN	NaN	NaN	NaN	NaN	NaN	25,51048	24,0141	NaN
PHKA1	25,06829	NaN	NaN	NaN	NaN	NaN	25,00873	23,95358	NaN	23,94315	NaN	NaN	NaN	24,07643	NaN	NaN	NaN	NaN	24,65911	NaN	NaN	23,11607	NaN	NaN	NaN
GRB7	NaN	25,52367	NaN	26,66058	NaN	NaN	NaN	NaN	28,40036	NaN	NaN	NaN	24,85067	NaN	23,57531	NaN	NaN	NaN	NaN	NaN	NaN	NaN	NaN	23,79035	23,35232
ECM29	NaN	NaN	NaN	NaN	24,29502	NaN	NaN	25,00612	NaN	NaN	NaN	NaN	NaN	NaN	NaN	NaN	NaN	NaN	NaN	24,69721	NaN	NaN	NaN	NaN	NaN
GALNT7	NaN	NaN	NaN	NaN	NaN	NaN	NaN	NaN	24,37352	NaN	NaN	NaN	NaN	NaN	NaN	NaN	NaN	NaN	NaN	NaN	24,1747	25,28061	NaN	NaN	NaN
OTUB1	NaN	25,73407	NaN	NaN	NaN	NaN	NaN	27,86012	NaN	NaN	NaN	NaN	NaN	NaN	NaN	25,24329	26,00112	NaN	24,55262	NaN	24,71931	NaN	25,60589	NaN	24,76436
SDSL	NaN	NaN	NaN	NaN	NaN	NaN	NaN	NaN	25,63392	NaN	24,6888	NaN	NaN	NaN	NaN	NaN	NaN	NaN	NaN	NaN	NaN	NaN	24,38824	NaN	NaN
CACYBP	NaN	23,23155	NaN	NaN	NaN	NaN	NaN	NaN	NaN	23,80607	24,50833	NaN	24,14725	NaN	NaN	24,36986	NaN	NaN	24,68024	24,06069	NaN	NaN	NaN	NaN	24,75241
NANS	NaN	NaN	NaN	NaN	NaN	NaN	NaN	24,16584	NaN	NaN	NaN	NaN	NaN	NaN	NaN	NaN	NaN	NaN	NaN	25,78925	NaN	NaN	NaN	NaN	NaN
ARFGEF2	NaN	NaN	NaN	24,4729	NaN	24,89832	25,37132	NaN	25,63326	25,10984	NaN	24,24402	NaN	23,76263	NaN	23,74016	NaN	25,90768	24,27234	25,67481	NaN	23,86022	NaN	25,00573	NaN

References

1. Thery, C. *et al.* Minimal information for studies of extracellular vesicles 2018 (MISEV2018): a position statement of the International Society for Extracellular Vesicles and update of the MISEV2014 guidelines. *J Extracell Vesicles* **7**, 1535750 (2018).
