# Supplementary material for: Proteomic profiling of extracellular vesicles allows for human breast cancer subtyping
Source: Commun Biol. 2019 Sep 3;2:325. doi: 10.1038/s42003-019-0570-8 (PMC6722120; doi:10.1038/s42003-019-0570-8)
Supplement: Supplementary file 2 — Description of Additional Supplementary Items [file 42003_2019_570_MOESM2_ESM.pdf]

Description of additional supplementary items

Supplementary Data 1. EV Protein groups file. List of the quantified EV proteins across the different EV samples, including the differentially expressed protein groups identified by ANOVA between the different EV subtypes.

Supplementary Data 2. EV phosphoproteome. List of all phosphosites quantified across all EVs with a localization probability >0.75. List of all quantified phosphosites of the EV phosphorylated kinases.

Supplementary Data 3. WCL Protein groups file. List of the quantified proteins across the different cell lines.
