# Supplementary material for: Proteomic profiling of extracellular vesicles allows for human breast cancer subtyping
Source: Commun Biol. 2019 Sep 3;2:325. doi: 10.1038/s42003-019-0570-8 (PMC6722120; doi:10.1038/s42003-019-0570-8)
Supplement: Supplementary file 6 — Reporting Summary [file 42003_2019_570_MOESM6_ESM.pdf]

nature research

Corresponding author(s): Maarten Altelaar (m.altelaar@uu.nl); Wei Wu (w.wu1@uu.nl)

Last updated by author(s): 08-02-2019

Reporting Summary

Nature Research wishes to improve the reproducibility of the work that we publish. This form provides structure for consistency and transparency in reporting. For further information on Nature Research policies, see Authors & Referees and the Editorial Policy Checklist .

Statistics

For all statistical analyses, confirm that the following items are present in the figure legend, table legend, main text, or Methods section.

n/a	Confirmed	
☐	☒	The exact sample size (*n*) for each experimental group/condition, given as a discrete number and unit of measurement
☐	☒	A statement on whether measurements were taken from distinct samples or whether the same sample was measured repeatedly
☐	☒	The statistical test(s) used AND whether they are one- or two-sided<br>*Only common tests should be described solely by name; describe more complex techniques in the Methods section.*
☐	☒	A description of all covariates tested
☐	☒	A description of any assumptions or corrections, such as tests of normality and adjustment for multiple comparisons
☐	☒	A full description of the statistical parameters including central tendency (e.g. means) or other basic estimates (e.g. regression coefficient) AND variation (e.g. standard deviation) or associated estimates of uncertainty (e.g. confidence intervals)
☒	☐	For null hypothesis testing, the test statistic (e.g. *F*, *t*, *r*) with confidence intervals, effect sizes, degrees of freedom and *P* value noted<br>*Give P values as exact values whenever suitable.*
☒	☐	For Bayesian analysis, information on the choice of priors and Markov chain Monte Carlo settings
☒	☐	For hierarchical and complex designs, identification of the appropriate level for tests and full reporting of outcomes
☒	☐	Estimates of effect sizes (e.g. Cohen's *d*, Pearson's *r*), indicating how they were calculated

Our web collection on statistics for biologists contains articles on many of the points above.

Software and code

Policy information about availability of computer code

Data collection	Mass spectrometry data acquisition and processing was performed using Thermo Scientific Xcalibur Software (version 4.1).
Data analysis	All raw MS files were searched with the MaxQuant software (versions 1.5.8.3 and 1.6.2.3). All data were analyzed using the Perseus software (version 1.6.1.1), Excel 2016, GraphPad Prism 7, the R statistical software (version 3.5.1). Gene ontology (GO) analyses were performed with Database for Annotation, Visualization and Integrated Discovery (DAVID) v6.8. Gene set enrichment analysis (GSEA) was performed using Broad GSEA version 3.0, using gene set collections from the Molecular Signatures database (MSigDB) v6.1. Interaction and pathway enrichment of phosphorylated kinases were performed using Cytoscape software platform (version 3.6.1) with the PhosphoPath plug-in. Functional enrichment analysis of the TNBC and HER2-positive EV subtype signatures using the online ToppCluster tool.

For manuscripts utilizing custom algorithms or software that are central to the research but not yet described in published literature, software must be made available to editors/reviewers. We strongly encourage code deposition in a community repository (e.g. GitHub). See the Nature Research guidelines for submitting code & software for further information.

Data

Policy information about availability of data

All manuscripts must include a data availability statement. This statement should provide the following information, where applicable:

- Accession codes, unique identifiers, or web links for publicly available datasets
- A list of figures that have associated raw data
- A description of any restrictions on data availability

All mass spectrometry proteomics data have been deposited to the ProteomeXchange Consortium via the PRIDE partner repository with the dataset identifier PXD012162 (p. 19, manuscript).

Field-specific reporting

Please select the one below that is the best fit for your research. If you are not sure, read the appropriate sections before making your selection.

- [x] Life sciences
- [ ] Behavioural & social sciences
- [ ] Ecological, evolutionary & environmental sciences

For a reference copy of the document with all sections, see nature.com/documents/nr-reporting-summary-flat.pdf

Life sciences study design

All studies must disclose on these points even when the disclosure is negative.

Sample size	10 cell lines were used for EV isolation in the discovery set. For validation, 20 frozen serum samples were available.
Data exclusions	No data were excluded from analysis.
Replication	Cell line-derived EVs were isolated from four biological replicates (n=4) per cell line. Therefore, the EV proteome and EV phosphoproteome analysis was performed on 4 biological replicates. Whole proteome was measured in three biological replicates.
Randomization	Order in mass spect analysis
Blinding	N/A.

Reporting for specific materials, systems and methods

We require information from authors about some types of materials, experimental systems and methods used in many studies. Here, indicate whether each material, system or method listed is relevant to your study. If you are not sure if a list item applies to your research, read the appropriate section before selecting a response.

Materials & experimental systems

n/a	Involved in the study	
[ ]	[x]	Antibodies
[ ]	[x]	Eukaryotic cell lines
[x]	[ ]	Palaeontology
[x]	[ ]	Animals and other organisms
[x]	[ ]	Human research participants
[x]	[ ]	Clinical data

Methods

n/a	Involved in the study	
[x]	[ ]	ChIP-seq
[x]	[ ]	Flow cytometry
[x]	[ ]	MRI-based neuroimaging

Antibodies

Antibodies used	mouse monoclonal antibody CD81 (5A6, Santa Cruz Biotechnology, Santa Cruz CA). Catalog # sc-23962. Lot # G2415.
Validation	PMID: # 29985392 Torralba, D. et al. 2018. Nat Commun. 9: 2658 (reference obtained from Santa Cruz website). Raised against OCI-LY8 cells. Recommended for detection of CD81 of mouse, rat and human origin by WB, IP, IF and FCM (website information).

Eukaryotic cell lines

Policy information about cell lines

Cell line source(s)	All cell lines were obtained from the ATCC.
Authentication	Not applicable.
Mycoplasma contamination	All cell lines used in this study were tested negative for mycoplasma contamination.
Commonly misidentified lines (See ICLAC register)	None.
